# Effectiveness of Mindfulness‐Based Interventions for Fear of Childbirth Among Pregnant Women Planned for Normal Birth: A Systematic Review and Meta‐Analysis

**DOI:** 10.1111/wvn.70101

**Published:** 2026-01-19

**Authors:** Ruohan Wang, Yinge Wang, Yue Huang, Ka Ming Chow

**Affiliations:** ^1^ The Nethersole School of Nursing, Faculty of Medicine The Chinese University of Hong Kong Shatin Hong Kong China; ^2^ Department of Obstetrics and Gynecology, Guangdong Provincial Key Laboratory of Major Obstetric Diseases; Guangdong Provincial Clinical Research Center for Obstetrics and Gynecology; Guangdong‐Hong Kong‐Macao Greater Bay Area Higher Education Joint Laboratory of Maternal‐Fetal Medicine The Third Affiliated Hospital of Guangzhou Medical University Guangzhou China; ^3^ Department of Nursing, Guangzhou Women and Children's Medical Center Guangzhou Medical University Guangzhou China

**Keywords:** fear of childbirth, meta‐analysis, mindfulness, normal birth, systematic review

## Abstract

**Background:**

Fear of childbirth is common among pregnant women. Mindfulness‐based interventions have been used widely in obstetrics. However, the evidence of the effects on fear of childbirth is controversial.

**Aims:**

To evaluate the effects of mindfulness‐based interventions on fear of childbirth, pain catastrophising, labour pain intensity, use of pain relief medication, mode of delivery and duration of labour among pregnant women planned for normal delivery.

**Methods:**

In this systematic review, 10 databases were searched from inception to 7 November 2024. Randomized controlled trials implementing mindfulness‐based interventions for fear of childbirth or related outcomes were included. Two reviewers assessed the methodological quality and certainty of evidence independently. Standardized mean difference (SMD), risk ratio (RR), and 95% confidence intervals (CI) were used to evaluate effect sizes.

**Results:**

A total of 16 studies from 15 trials were included. Based on very low to moderate certainty of evidence, mindfulness‐based interventions were effective in decreasing fear of childbirth both immediately post‐intervention and within 6‐week postpartum (SMD: −0.72; 95% CI −0.89, −0.55; SMD: −0.63; 95% CI −0.91, −0.35, respectively), labour pain intensity (SMD: −1.22; 95% CI −2.07, −0.37), caesarean section rate (RR: 0.58; 95% CI 0.36, 0.93), and total duration of labour (SMD: −1.03; 95% CI −1.34, −0.72), and improving mindfulness level both immediately post‐intervention and within six‐week postpartum (SMD: 0.48; 95% CI 0.31, 0.66; SMD: 0.50; 95% CI 0.27, 0.73, respectively), but had no significant effect on pain catastrophising (SMD: −0.36; 95% CI −0.72, 0.01) and epidural analgesia use rate (RR: 0.77; 95% CI 0.57, 1.03).

**Linking Evidence to Action:**

Mindfulness‐based interventions have potential effects on reducing fear of childbirth and promoting labour‐related outcomes. These interventions might be an effective approach in obstetrics clinical practice to enhance the pregnancy and labour experiences among pregnant women.

**Trial Registration:**

PROSPERO registration number: CRD42024610793

## 1 Introduction

Childbirth is an important experience for women of reproductive age. During this period, women may experience complex emotions. Fear of childbirth (FOC), a distressing condition related to delivery, is common among pregnant women, especially first‐time mothers (Elsharkawy et al. 2024). The prevalence of FOC among pregnant women varies across countries, cultural backgrounds, and measurements, with severe FOC ranging from 3.7% to 43% (O'Connell et al. 2021) and moderate or above level of FOC exceeding 70% (Demšar et al. 2018). The level of FOC can range from mild, moderate, high to severe, which can interfere with one's daily life (Elsharkawy et al. 2024).

Actually, FOC has physical and psychological consequences. According to the fear‐tension‐pain cycle (Mozingo 1978), fear can activate the sympathetic nervous system, producing muscle tensions, causing the perception of pain during labour. In addition, excessive sympathetic activity caused by FOC and labour pain can result in incoordinate and decreased uterine contractions, which can prolong labour (Zagami et al. 2015). Meanwhile, prolonged labour has emerged as a primary indicator for instrumental delivery or caesarean section (CS) (Issac et al. 2023). Women with a high level of FOC are likely to avoid a natural delivery and request an elective CS or pharmacological pain relief, such as epidural analgesia (EA), before experiencing labour pain (Veringa‐Skiba et al. 2016). However, unnecessary CS and EA may elevate the risk of complications (Smith et al. 2019; Zuarez‐Easton et al. 2023). Furthermore, previous literature has indicated that FOC is highly related to pain catastrophising, characterized by an exaggerated focus on pain and negative evaluations of coping abilities (Whitburn et al. 2014).

Mindfulness entails the process of openly attending to one's present‐moment experience with awareness (Creswell 2017). Mindfulness‐based interventions (MBIs) have demonstrated effectiveness in managing various emotional disorders. In the field of obstetrics, MBIs have been used widely among pregnant women. However, the results of the effect of such interventions on FOC and related outcomes are controversial (Duncan et al. 2017; Wang, Sun, et al. 2023). Moreover, previous systematic reviews on such research have limitations, such as a lack of a systematic search of studies on FOC (Wang et al. 2024) or the inclusion of only FOC and self‐efficacy outcomes and neglect of other related outcomes (Abdolalipour et al. 2023). Given the inconsistent and inadequate evidence, this systematic review aims to evaluate the potential effects of MBIs on FOC, pain catastrophising, labour pain intensity, use of pain relief medication, mode of delivery and duration of labour among pregnant women planned for normal delivery.

## 2 Methods

This study followed the Cochrane Handbook for Systematic Reviews of Interventions and the guideline for reporting systematic reviews (Preferred Reporting Items for Systematic Reviews and Meta‐analyses [PRISMA]) (Page et al. 2021). The protocol of this systematic review was registered on November 6, 2024 in the International Prospective Register of Systematic Reviews.

### 2.1 Study Selection

Randomized controlled trials (RCTs) published in peer‐reviewed journals that met the following eligibility criteria were selected for this review: (1) the participants were pregnant women with a singleton pregnancy planned for a vaginal delivery, without complications or comorbidities; (2) the participants in the intervention group were provided with MBIs that included mindfulness as a core component; (3) the participants in the control group received other types of comparisons, such as usual care, attention control, or active control; and (4) FOC was the primary outcome, and pain catastrophising, mindfulness level, labour pain intensity, use of pain relief medication, mode of delivery, and duration of labour were the secondary outcomes, which were assessed within 6 weeks postpartum. The exclusion criteria were papers that were protocols, clinical trial registrations, conference abstracts, and other types of publications that lacked complete data.

### 2.2 Search Strategies

A systematic search was conducted in 10 databases, specifically six English databases MEDLINE (via Pubmed), Embase (via Ovid), the Cochrane Central Register of Controlled Trials (CENTRAL), the Cumulative Index to Nursing and Allied Health Literature (CINAHL), PsycINFO (via Ovid), dissertations and theses (via Proquest Dissertations & Theses, ProQuest Dissertations & Theses Global: The Humanities and Social Sciences Collection, and ProQuest Dissertations & Theses Global A&I: The Sciences and Engineering Collection); and four Chinese databases, the China National Knowledge Infrastructure (CNKI), the Wanfang Database, the Chinese Scientific Journals Database (VIP) and Chinese BioMedical Literature Database (CBM, via SinoMed), from inception to 7 November 2024. Keywords such as “parturition”, “delivery, obstetric”, “pregnancy”, “childbirth”, “labour”, “mindfulness”, “meditation” and “fear” were used to identify the relevant studies. The detailed search strategies are presented in Table S1. Only publications in English or Chinese were retrieved, and no restrictions were placed on the publication date. Additionally, the references of the included studies were searched manually, and the original authors of protocols, clinical trial registrations and conference abstracts were contacted to obtain the complete data or full text, if available.

### 2.3 Screening and Quality Appraisal

After removing duplicated records, two reviewers independently screened all retrieved records according to title and abstract, followed by a full‐text review based on the inclusion and exclusion criteria. Discrepancies were resolved through discussion with a third reviewer. The methodological quality was evaluated independently by two reviewers using the Cochrane risk‐of‐bias tool for randomized trials version 2 (RoB2), which assesses five aspects of bias: randomization process, intended interventions, missing outcome data, measurement of the outcome, and the selection of the reported result. Each aspect was rated as having a low risk of bias, some concerns, or a high risk of bias (Higgins et al. 2024). Any discrepancies were discussed by these two reviewers, and a third reviewer was consulted if consensus could not be reached.

### 2.4 Certainty of Evidence

The certainty of evidence for each outcome was assessed independently by two reviewers using the Grading of Recommendations Assessment, Development and Evaluation (GRADE) method via GRADE profiler Guideline Development Tool (GRADEpro GDT) (Ryan 2016), which contains five criteria: risk of bias, inconsistency, indirectness, imprecision, and publication bias. Besides, the certainty of evidence was upgraded based on large effect, dose–response gradient, and plausible confounding. Outcomes were classified into four levels of certainty: high, moderate, low, or very low certainty. Any discrepancies were discussed by these two reviewers, and a third reviewer was consulted if consensus could not be reached.

### 2.5 Data Extraction

Two reviewers independently extracted data from the included studies. The extracted information included publication information (authors, publication year, country or region), participant characteristics (inclusion and exclusion criteria, and sample size), intervention details (settings, contents, format, duration, facilitator, adherence and fidelity), control condition, outcomes and results. Any discrepancies were discussed by these two reviewers, and a third reviewer was consulted if consensus could not be reached.

### 2.6 Data Analysis

The effectiveness of the interventions was assessed at two timepoints: immediately post‐intervention and within 6 weeks post‐delivery. Meta‐analysis was performed when the same outcomes were measured at the same timepoints. For the continuous variables, the pooled effect size was evaluated by using the standardized mean difference (SMD) and 95% confidence interval (CI), and an effect size of 0.2, 0.5, or 0.8 was considered to be a small, medium, or large effect, respectively. For the dichotomous variables, the risk ratio (RR) and 95% CI were used, and an effect size of 1.22 (or 0.82), 1.86 (or 0.54), or 3.00 (or 0.33) was regarded as small, medium, or large, respectively (Olivier et al. 2017). Heterogeneity was assessed with Cochran's *Q* test and the *I* ^2^ statistic, and *p* ≥ 0.1 in Cochran's *Q* test and *I* ^2^ value < 50% were regarded as acceptable, for which a fixed‐effects model was used. Otherwise, a random‐effects model was employed.

Moreover, sensitivity analysis was conducted by excluding the studies with a high risk of bias, and subgroup analysis was performed on the studies that conducted intention‐to‐treat (ITT) or per‐protocol (PP) analysis, with different baseline levels of FOC, and applied different control conditions to explore the different effects. The number of eligible studies for all the outcomes was fewer than 10; thus, publication bias was not assessed. All the analyses were performed with RevMan 5.3. Narrative synthesis was performed for the outcomes with insufficient or highly heterogeneous data.

## 3 Results

### 3.1 Search Results

**FIGURE 1:**
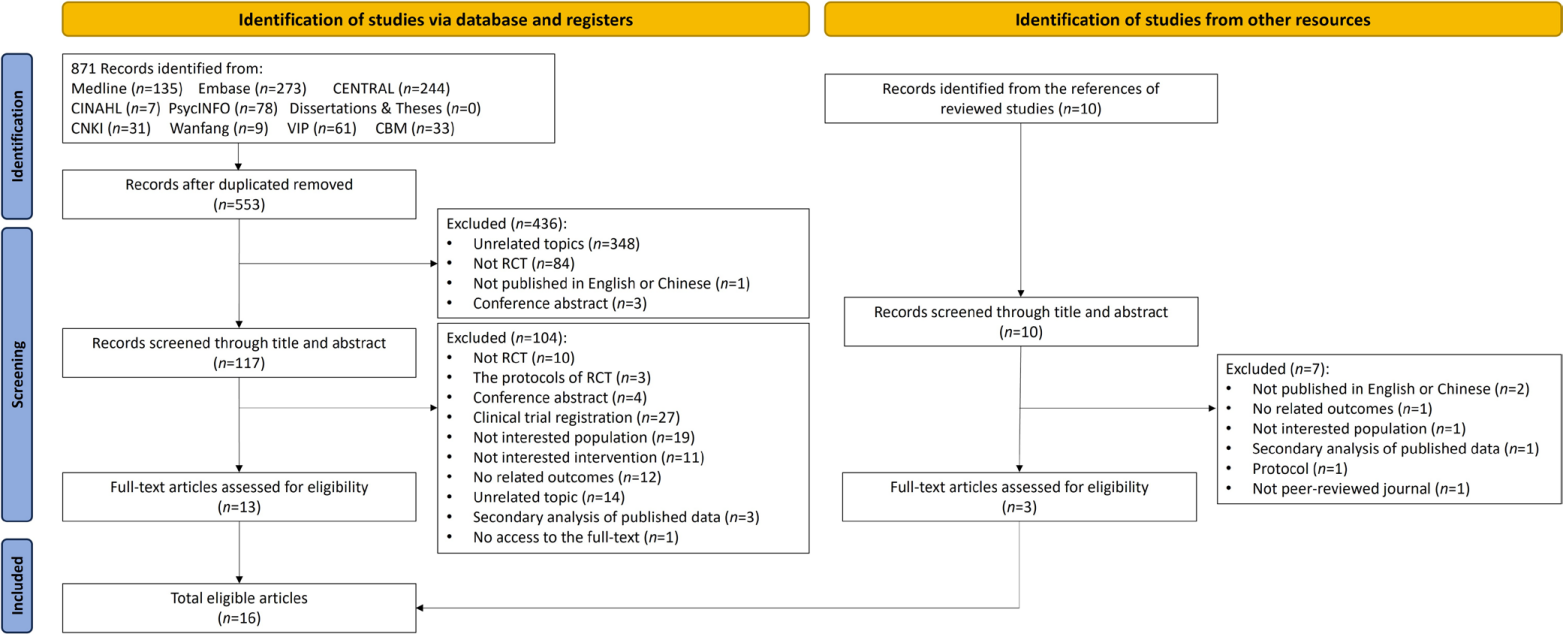
Study selection flow chart (PRISMA).

A total of 871 records were identified from 10 databases. After removal of duplicates (*n* = 318), 553 records were retained. Following title and abstract screening, 117 records were selected for full‐text reviews, among which 104 were excluded. Additionally, three articles were identified through manual searches of the reference lists. Ultimately, 16 studies from 15 trials were included in this systematic review. Among them, two publications reporting different outcomes were generated from one trial. The PRISMA flowchart of study selection is presented in Figure 1.

### 3.2 Study Characteristics

The study characteristics are summarized in Table 1. The 15 trials were conducted across eight countries or regions, including mainland China (*n* = 7), Iran (*n* = 2), Australia (*n* = 1), Hong Kong (*n* = 1), the United States (*n* = 1), Turkey (*n* = 1), Taiwan (*n* = 1), and the Netherlands (*n* = 1). These trials were published between 2017 and 2024, and had sample sizes per study ranging from 20 to 141 participants. Among the 1278 participants, 641 were assigned to the intervention group, while 637 were allocated to the control group.

**TABLE 1:** Characteristics of the included studies (*N* = 16).

Study no.	Study ID	Design	Setting	Sample	Intervention										Control	Measures	Results
					Theory	Contents	Components	Format	Duration	Total hours	Number of sessions	Facilitator	Adherence	Fidelity			
1	Beattie et al. 2017	RCT	Australia	Women with a singleton pregnancy at 24–28 weeks of gestation (*N* = 20). Exclude women scored 13 or higher in EDPS, which indicates an increased risk for depression	The co‐emergence model	MiPP: (1) mindfulness practice and discussion; (2) birthing suite tour	Childbirth education, mindfulness; emotional support	Face‐to‐face, group‐based	8 weeks	~16 h	8	A midwife researcher/investigator trained in mindfulness‐based approaches	50.0% received intervention; 33.3% completed 4 or more sessions; Attrition rate was 62.5%; Reasons for dropout: exhaustion, health condition, felt the course was more suitable for first‐time mothers	No information	Active control: 8‐week group PSP	Mindfulness (post‐intervention): MAAS Mindfulness (postpartum, 6‐week post‐intervention): MAAS CS rate (%)	No significant difference was found between the intervention and control groups
2	Zhang et al. 2023	RCT	Hong Kong	Women with a singleton pregnancy in the 2nd and 3rd trimesters (*N* = 183)	—	(1) Mindfulness training; (2) self‐practice: mindfulness meditation at home for 30 to 45 min each day	Childbirth education; mindfulness; emotional support; coping skill training	Group‐based (group size: 18)	9 weeks	24.75 h	9	Two trained MBCP instructors	68.1% completed 4 or more sessions; Attrition rate (intervention group) was 36.2%	Continual supervision of facilitator and fidelity manual	Active control: 9‐week ACES	Pain catastrophising (post‐intervention): PCS Mindfulness (post‐intervention): FFMQ CS rate (%) EA use (%) Pain medication pethidine use (%) Duration of labour (the first, second, and third stage of labour)	The intervention group reported a higher mindfulness level post‐intervention (*p* = 0.004) than the control group. No significant difference was found in other outcomes
3	Wang, Zhang, et al. 2023	RCT	Mainland China	Women with a single pregnancy at 20–32 weeks of gestation (*N* = 83)	—	(1) Mindfulness training, labour pain cognitive education, and pain management; (2) self‐practice: practice at home for 30–40 min per day, 6 days a week	Childbirth education; mindfulness; coping skill training	Face‐to‐face, group‐based	4 weeks	14 h	4	Two MBCP teachers	Attrition rate (intervention group) was 8.3%; no information about intervention completion	No information	Active control: an online childbirth education course, lasting about 5–10 min per day, within 21 days	Fear of childbirth (FOC, post‐intervention): W‐DEQ version A (W‐DEQ‐A) FOC (3‐day postpartum): W‐DEQ version B (W‐DEQ‐B) Mindfulness (immediately post‐intervention): FFMQ Mindfulness (42‐day postpartum): FFMQ	The intervention group reported a lower FOC level and higher mindfulness level post‐intervention and postpartum (all *p* < 0.05) than the control group
4	Wang, Sun, et al. 2023	RCT	Mainland China	Women with a singleton pregnancy at 20–32 weeks of gestation (*N* = 95)	—	(1) Two‐day on‐site simplified version of the MBCP course over a weekend; (2) 21‐day online course via the WeChat applet with recorded audio, which lasted for 5–35 min per day	Mindfulness; coping skill training	Face‐to‐face (2 days), group‐based (group size: 30), online (21 days)	2 days	12 h	2	Two MBCP teachers	93.1% completed intervention; Attrition rate (intervention group) was 6.8%; reasons for dropout: lack of outcome measurements	No information	Active control: pregnancy and childbirth education course for 21 days, approximately 5–10 min per day	Mindfulness (post‐intervention): FFMQ	No significant difference was found between the intervention and control groups
5	Duncan et al. 2017	RCT	United States	Women with low‐risk singleton pregnancies in their third trimester (*N* = 29)	—	MBCP: (1) formal mindfulness meditation; (2) an enquiry practice between partners exploring fear; (3) handouts and guided audio materials for self‐practice use	Mindfulness; coping skill training; emotional support	Group‐based	2.5 days	18 h	1	Professionally certified MBCP instructors	80.0% completed intervention; 86.7% completed 1/2 of intervention; No dropout; Reasons: transportation, schedule conflict, uncomfortable about the intervention	No information	Active control: childbirth courses of comparable length and quality to the intervention group	FOC (post‐intervention): W‐DEQ‐A FOC (6‐week postpartum): W‐DEQ‐B Pain Catastrophising (post‐intervention): PCS Mindfulness (post‐intervention): FFMQ Pain intensity (Average): VAS EA rate (%) Pain medication use (%): narcotic	No significant difference was found between the intervention and control groups
6	Gökbulut et al. 2024	RCT	Turkey	Women with singleton pregnancy at 12–24 weeks of gestation (*N* = 64). Exclude women who had attended regular mind–body practices	—	(1) Mindfulness and meditation techniques; (2) self‐practice: home assignments (audio recording) to be completed in the form of repetitions of meditations.	Mindfulness	Online, individual or group, determined by participants' availability	4 weeks	5.3–8 h	8	A researcher who had received MBSR training	Attrition rate (intervention group) was 5.9%; no information about intervention completion	No information	Usual care	FOC (post‐intervention): subscale of PRAQ‐R2	The intervention group reported a lower FOC level post‐intervention (*p* < 0.001) than the control group
7	Oskoui et al. 2023	RCT	Iran	Primiparous women with gestational ages of 32–34 weeks and a cephalic singleton pregnancy (*N* = 64)	—	(1) Counseling sessions with mindfulness practice; (2) self‐practice: 40‐60 min every day	Mindfulness; emotional support	Online (4 sessions) and face‐to‐face (4 sessions)	8 weeks	6 h	8	A senior student of midwifery counseling and mindfulness‐based counseling	100.0% completed all intervention, and no dropout	No information	Usual care	Pain intensity (Before 6 cm dilatation): VAS Pain intensity (After 6 cm dilatation): VAS	No significant difference was found between the intervention and control groups
8	Azh et al. 2021	RCT	Iran	Primiparous women with a single pregnancy at 20–22 weeks of gestation (*N* = 112). Exclude women who had experience of yoga or meditation	—	Mindfulness training, between 20–22 weeks and 36 weeks	Mindfulness	—	—	8 h	8	Midwifes with mindfulness training, a clinical psychologist	100.0% received intervention; Attrition rate (intervention group) was 30.9%; Reasons: medical reasons and transfer to other hospitals	No information	Usual care	Pain intensity (4–6 cm dilatation): VAS Pain intensity (7–8 cm dilatation): VAS Pain intensity (9–10 cm dilatation): VAS	The intervention group reported a lower pain intensity at three timepoints (*p* = 0.001) than the control group
9	Kuo et al. 2022	RCT	Taiwan	Women with a singleton pregnancy at 12–24 weeks of gestation, with a score of ≥ 7 points on the FOC VAS (*N* = 106)	The theory of mindfulness‐based stress reduction, the principles of adult learning theory	(1) Childbirth sessions: pregnancy and labour process, intrapartum care, pain relief during labour, breastfeeding, newborn care, and postpartum care. (2) Mindfulness sessions: mindfulness practice	Childbirth education; mindfulness; coping skill training	Group‐based	8 weeks	16 h	8	An experienced childbirth practitioner and a certified mindfulness lecturer	86.8% completed intervention; attrition rate (intervention group) was 13.2%; Reasons for dropout: medical reasons and transfer to another clinic	No information	Usual care	FOC (post‐intervention, 36 weeks of gestation): W‐DEQ‐A FOC (1‐week postpartum): W‐DEQ‐B Mindfulness (post‐intervention, 36 weeks of gestation): MAAS Mindfulness (1‐week postpartum): MAAS	The intervention group reported a lower FOC both post‐intervention (95% CI: −20.5, −6.3) and postpartum (95% CI: −30.2, −14.3) than the control group. No significant difference of mindfulness level was found between groups
10	Van der Meulen et al. 2023	RCT	Netherlands	Women with gestational ages of 16–26 weeks and a high level of FOC (W‐DEQ‐A ≥ 66) (*N* = 141)	A theoretical model of avoidance and participation in pregnancy, birth and the postpartum period	(1) Mindfulness meditation practice and enquiry; (2) psychobiological processes in the perinatal period for women, newborns, and the family. (3) self‐practice: meditation practices at home for 30 min each day.	Childbirth education; mindfulness; coping skill training; emotional support	Face‐to‐face, group‐based	9 weeks	27 h	9	Experienced midwives certified in MBCP	85.3% received > 3 sessions; Reasons: personal issues, medical reasons, and no reasons; Attrition rate (intervention group) was 25.3%	Intervention was recorded and supervised	Enhanced care as usual: two 90‐min consultation sessions on FOC to the usual care	FOC (two‐four weeks after birth): W‐DEQ‐B Pain intensity (Average): VAS	No significant difference was found between the intervention and control groups
11	Veringa‐Skiba, de Bruin, et al. 2022; Veringa‐Skiba, Ziemer, et al. 2022	RCT	Netherlands													FOC (post‐intervention): W‐DEQ‐A Pain catastrophising (post‐intervention): CLP CS rate (%) EA rate (%)	The intervention group reported a lower FOC level (*t* = −2.74, *p* = 0.01), pain catastrophising (*t* = −3.75, *p* < 0.001), CS rate (*χ* ^2^ = 6.860, *p* = 0.009) and EA rate (*χ* ^2^ = 4.854, *p* = 0.028) than the control group.
12	Gao et al. 2024	RCT	Mainland China	Primiparous women with a singleton pregnancy at 16–32 weeks of gestation and a high level of FOC (W‐DEQ‐A ≥ 66) (*N* = 114). Exclude women who had experience of mindfulness or meditation	—	(1) Mindfulness training; (2) Childbirth education: labour process and labour pain, neonatal care; (3) self‐practice: mindfulness skill practice for continuously 21 days	Childbirth education; mindfulness; coping skill training	Face‐to‐face (2 days), online (21 days)	2 days	—	2	Midwives	76.3% completed intervention; dropout rate was 27.1% Reasons for dropout: complications, no reason	No information	Usual care	FOC (post‐intervention): W‐DEQ FOC (3‐day postpartum): W‐DEQ Mindfulness (post‐intervention): FFMQ Mindfulness (42‐day postpartum): FFMQ CS rate (%) EA rate (%) Duration of labour (the first, second, and third stage of labour)	The intervention group reported lower levels of FOC post‐intervention (95% CI: −17.03, −5.91) and postpartum (95% CI: −19.63, −7.05), higher levels of mindfulness post‐intervention (95% CI: 4.52, 12.78) and postpartum (95% CI: 1.86, 9.51), and a lower EA rate (*χ* ^2^ = 5.433, *p* = 0.020) than the control group. No significant difference was reported in other outcomes.
13	Guo and Lei 2022	RCT	Mainland China	Primiparous women with a singleton pregnancy and no complications or comorbidities (*N* = 100)	—	Mindfulness practice	Childbirth education; mindfulness; coping skill training; emotional support	Face‐to‐face and online, group‐based	8 weeks	—	8	Midwives	No dropout; no information about intervention completion	No information	Usual care	FOC (before birth): CAQ Pain intensity (average): Numerical rating scale (NRS) CS rate (%) Duration of labour (total)	The intervention group reported a lower FOC level (*t* = −5.242, *p* < 0.010), pain intensity level (*t* = −4.331, *p* < 0.010) and CS rate (*χ* ^2^ = 7.143, *p* < 0.010), and a shorter duration of labour (*t* = −5.921, *p* < 0.010) than the control group.
14	Li et al. 2021	RCT	Mainland China	Women with a singleton pregnancy at over 35 weeks of gestation (*N* = 100)	—	(1) Mindfulness practice from recruitment to childbirth; (2) self‐practice: practice mindfulness skill at home; (3) 30 min before childbirth	Mindfulness; coping skill training	Face‐to‐face and online, individual	From recruitment to childbirth	—	5	Midwives	No information about intervention completion and dropout	No information	Usual care	Pain intensity (the first, second, and third stages of labour): VAS Mindfulness (post‐intervention): FFMQ	The intervention group reported a lower pain intensity level during the first, second, and third stages of labour (*t* = 5.970, *p* < 0.001; *t* = 3.704, *p* < 0.001; *t* = 3.773, *p* < 0.001, respectively) and a higher mindfulness level (*t* = 3.362, *p* = 0.001) than the control group.
15	Pan et al. 2020	RCT	Mainland China	Primiparous women with a singleton pregnancy (*N* = 60)	—	(1) Mindfulness training; (2) self‐practice: 20 min every day	Mindfulness	Online, group‐based	8 weeks	3 h 45 min	8	Midwives	No dropout; no information about intervention completion	No information	Usual care	Pain intensity (the first, second, and third stages of labour): VAS Duration of labour (the first, second, and third stages of labour)	The intervention group reported a lower pain intensity level during the first, second, and third stages of labour (*t* = 12.489, *p* < 0.001; *t* = 2.906, *p* = 0.005; *t* = 3.688, *p* = 0.001, respectively), and a shorter duration of the first, second, and third stages of labour (*t* = 11.401, *p* < 0.001; *t* = 2.270, *p* = 0.027; *t* = 4.104, *p* < 0.001, respectively) than the control group.
16	Zhao et al. 2024	RCT	Mainland China	Primiparous women with a singleton pregnancy at 31–32 weeks of gestation (*N* = 80)	—	Mindfulness	Mindfulness	Group‐based	8 weeks	8 h	8	Midwives	No dropout; no information about intervention completion	No information	Usual care	FOC (post‐intervention): CAQ CS rate (%) Duration of labour (total)	The intervention group reported a lower FOC level (*t* = 3.840, *p* < 0.001) and CS rate (*χ* ^2^ = 4.114, *p* = 0.043), and a shorter total duration of labour (*t* = 3.874, *p* < 0.001) than the control group.

Abbreviations: ACES, Antenatal Childbirth Education and Support; CAQ, Childbirth Attitudes Questionnaires; CLP, Catastrophising Labor Pain; CS, Caesarean section; EA, Epidural analgesia; EDPS, Edinburgh Postnatal Depression Scale; FFMQ, Five Facet Mindfulness Questionnaire; FOC, fear of childbirth; MAAS, Mindfulness Attention Awareness Scale; MBCP, Mindfulness‐based childbirth and parenting; MiPP, Mindfulness in Pregnancy Program; NRS, Numerical rating scale; PCS, Pain Catastrophising scale; PRAQ‐R2, Pregnancy‐Related Anxiety Questionnaire‐Revision‐2; PSP, Pregnancy Support Programme; VAS, Visual analogue scale; W‐DEQ, Wijma Delivery Expectancy/Experience Questionnaire.

### 3.3 Characteristic of Participants

Six studies from five trials recruited participants during their second trimester (Azh et al. 2021; Beattie et al. 2017; Gökbulut et al. 2024; Kuo et al. 2022; Van der Meulen et al. 2023; Veringa‐Skiba, de Bruin, et al. 2022), four studies recruited participants in their third trimester (Duncan et al. 2017; Li et al. 2021; Oskoui et al. 2023; Zhao et al. 2024), other four studies recruited participants during both the second and third trimesters (Gao et al. 2024; Wang, Zhang, et al. 2023; Wang, Sun, et al. 2023; Zhang et al. 2023). Moreover, seven trials recruited only first‐time pregnant women (primiparous women) (Azh et al. 2021; Duncan et al. 2017; Gao et al. 2024; Guo and Lei 2022; Oskoui et al. 2023; Pan et al. 2020; Zhao et al. 2024). Furthermore, four studies from three trials recruited only women with a high level of FOC (Gao et al. 2024; Kuo et al. 2022; Van der Meulen et al. 2023; Veringa‐Skiba, de Bruin, et al. 2022).

### 3.4 Characteristics of Interventions and Controls

Regarding the intervention, four components, including mindfulness, childbirth education, coping strategies, and emotional support, were identified from the included studies. Mindfulness was a core component in all trials, which involved mindfulness education and skill training, with most requiring 5–60 min of self‐practicing. Additionally, seven studies from six trials included childbirth education, providing information regarding pregnancy and labour process, and intrapartum and postpartum care (Beattie et al. 2017; Gao et al. 2024; Guo and Lei 2022; Van der Meulen et al. 2023; Veringa‐Skiba, de Bruin, et al. 2022; Wang, Sun, et al. 2023; Zhang et al. 2023). Nine studies from eight trials also contained coping strategies, such as pain relief methods to assist participants in coping with labour pain (Duncan et al. 2017; Gao et al. 2024; Guo and Lei 2022; Li et al. 2021; Van der Meulen et al. 2023; Veringa‐Skiba, de Bruin, et al. 2022; Wang, Zhang, et al. 2023; Wang, Sun, et al. 2023; Zhang et al. 2023). Furthermore, eight studies from seven trials provided emotional support, such as practice to explore fear, mental health and even postnatal depression (Beattie et al. 2017; Duncan et al. 2017; Guo and Lei 2022; Kuo et al. 2022; Oskoui et al. 2023; Van der Meulen et al. 2023; Veringa‐Skiba, de Bruin, et al. 2022; Zhang et al. 2023). In addition, only four studies of three trials applied theoretical models to guide the intervention development (Beattie et al. 2017; Kuo et al. 2022; Van der Meulen et al. 2023; Veringa‐Skiba, de Bruin, et al. 2022).

For the intervention delivery format, six studies from five trials (Beattie et al. 2017; Gao et al. 2024; Van der Meulen et al. 2023; Veringa‐Skiba, de Bruin, et al. 2022; Wang, Zhang, et al. 2023; Wang, Sun, et al. 2023) implemented a face‐to‐face intervention, two trials (Gökbulut et al. 2024; Pan et al. 2020) conducted an online intervention and three trials (Guo and Lei 2022; Li et al. 2021; Oskoui et al. 2023) combined face‐to‐face and online interventions. The majority of studies conducted a group‐based intervention, with a group size ranging from 12 to 30 (Beattie et al. 2017; Duncan et al. 2017; Guo and Lei 2022; Kuo et al. 2022; Pan et al. 2020; Van der Meulen et al. 2023; Veringa‐Skiba, de Bruin, et al. 2022; Wang, Zhang, et al. 2023; Wang, Sun, et al. 2023; Zhang et al. 2023; Zhao et al. 2024), whereas one study conducted individual sessions (Li et al. 2021) and another study adopted a flexible individual or group‐based method based on the participants' availability (Gökbulut et al. 2024). Most of the trials employed the classic eight‐ or nine‐week mindfulness training design, with eight or nine sessions, whereas the other trials implemented short interventions, including 4 weeks with four (Wang, Sun, et al. 2023) or eight (Gökbulut et al. 2024) sessions, or intensive 2.5‐day (Duncan et al. 2017) or 2‐day (Gao et al. 2024; Wang, Zhang, et al. 2023) courses. The intervention duration ranged from 5.3 h to 27 h. Furthermore, midwives or nurses who received mindfulness training conducted most of the interventions, while in other interventions, mindfulness instructors (Beattie et al. 2017; Duncan et al. 2017; Gökbulut et al. 2024; Kuo et al. 2022; Wang, Zhang, et al. 2023; Wang, Sun, et al. 2023; Zhang et al. 2023) or psychologists (Azh et al. 2021) were involved in the intervention implementation.

Eight trials reported intervention completion rates, ranging from 33.3% to 100.0%. Due to various follow‐up timepoints across the trials, attrition rates were calculated based on the immediate post‐intervention timepoint, which ranged from 0.0% to 62.5%. The reported reasons for dropout included personal issues (exhaustion, schedule conflicts, and transportation), medical reasons (health conditions, complications, transfer to other hospitals), and other reasons related to the intervention (felt the intervention was not suitable and uncomfortable with the intervention). Furthermore, only two trials (three studies) reported strategies to ensure intervention fidelity, one applying continual supervision of the facilitator and fidelity manual (Zhang et al. 2023), and another reporting that the intervention was recorded and supervised (Van der Meulen et al. 2023; Veringa‐Skiba, de Bruin, et al. 2022).

Regarding the control conditions, seven studies from six trials employed active control, five providing childbirth education courses with comparable lengths to the intervention group (Beattie et al. 2017; Duncan et al. 2017; Wang, Zhang, et al. 2023; Wang, Sun, et al. 2023; Zhang et al. 2023), and one trial (two studies) providing “enhanced care as usual” by offering two 90‐min consultation sessions on FOC (Van der Meulen et al. 2023; Veringa‐Skiba, de Bruin, et al. 2022). The remaining studies provided usual care, including standard perinatal care, such as routine antenatal visits.

### 3.5 Methodological Quality of Included Studies

**FIGURE 2:**
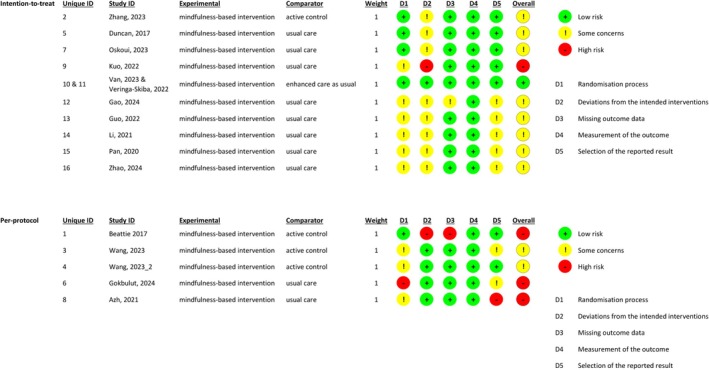
Methodological quality of included studies.

The methodological quality appraisal of the included studies is presented in Figure 2. Among the 10 trials conducting ITT analysis, only one trial (two studies) was rated with low risk of bias (Van der Meulen et al. 2023; Veringa‐Skiba, de Bruin, et al. 2022), one was identified as having a high risk of bias (Kuo et al. 2022), and the remaining trials were rated with some concerns (Duncan et al. 2017; Gao et al. 2024; Guo and Lei 2022; Li et al. 2021; Oskoui et al. 2023; Pan et al. 2020; Zhang et al. 2023; Zhao et al. 2024). Regarding the five studies conducting PP analysis, two were rated with some concerns (Wang, Zhang, et al. 2023; Wang, Sun, et al. 2023), while three were identified as having a high risk of bias (Azh et al. 2021; Beattie et al. 2017; Gökbulut et al. 2024).

### 3.6 Effects of Mind–Body Interventions on the Outcomes

#### 3.6.1 Fear of Childbirth

Regarding the immediate effect of the intervention, the meta‐analysis included 644 participants from seven studies. The results indicated that the FOC levels post‐intervention were significantly lower in the intervention group compared to those in the control group, with a medium effect size (SMD: −0.72, 95% CI −0.89, −0.55, *I* ^2^ = 9%) (Table 2), based on moderate certainty of evidence (Table 3, Figure S1a). Regarding the effect within 6 weeks post‐delivery, based on very low certainty of evidence (Table 3), the pooled results of 397 participants from five studies revealed a similarly significant decrease in FOC level in the intervention group compared to the control group, with a medium effect size (SMD: −0.63, 95% CI −0.91, −0.35, *I* ^2^ = 46%) (Table 2, Figure S1b). According to the forest plots, only one trial (Duncan et al. 2017), which delivered a 2.5‐day intervention without a childbirth education component, reported nonsignificant results immediately post‐intervention.

**TABLE 2:** Effects of mindfulness‐based interventions on the outcomes.

Outcomes	No. of study	Sample size	SMD/RR (95% CI)	*p*	*I* ^2^, %
FOC (post‐intervention)	7	644	‐0.72 (−0.89, −0.55)	< 0.001^a^	9
ITT analysis	6	561	−0.71 (−0.90, −0.51)	< 0.001^a^	22
PP analysis	1	83	−0.81 (−1.26, −0.37)	< 0.001^a^	—
FOC (within 6‐week postpartum)	5	397	−0.63 (−0.91, −0.35)	< 0.001^a^	46
ITT analysis	4	314	−0.67 (−1.02, −0.32)	< 0.001^a^	53
PP analysis	1	83	−0.46 (−0.89, −0.02)	0.040^a^	—
Pain catastrophising	3	274	−0.36 (−0.72, 0.01)	0.060	50
Mindfulness level (post‐intervention)	8	682	0.48 (0.31, 0.66)	< 0.001^a^	19
ITT analysis	5	484	0.47 (0.20, 0.74)	< 0.001^a^	51
PP analysis	3	198	0.47 (0.19, 0.75)	0.001^a^	0
Mindfulness level (within 6‐week postpartum)	3	303	0.50 (0.27, 0.73)	< 0.001^a^	0
ITT analysis	2	220	0.45 (0.18, 0.72)	0.001^a^	0
PP analysis	1	83	0.62 (0.18, 1.06)	0.006^a^	—
Pain intensity	5	389	−1.22 (−2.07, −0.37)	0.005^a^	93
CS rate	6	533	0.58 (0.36, 0.93)	0.020^a^	40
ITT analysis	5	514	0.53 (0.31, 0.91)	0.020^a^	48
PP analysis	1	19	0.97 (0.30, 3.18)	0.960	—
EA rate	4	329	0.77 (0.57, 1.03)	0.070	34
Duration of labour	2	180	−1.03 (−1.34, −0.72)	< 0.001^a^	0

Abbreviations: CS, Caesarean section; EA, Epidural analgesia; FOC, fear of childbirth; ITT, intention‐to‐treat; PP, per‐protocol.

^a^ *p* < 0.05.

**TABLE 3:** Certainty of evidence.

Outcomes	Anticipated absolute effects^f^ (95% CI)		Relative effect (95% CI)	No of participants (studies)	Certainty of the evidence (GRADE)
	Risk with control group	Risk with mindfulness‐based intervention			
FOC (post‐intervention)	—	SMD 0.72 SD lower (0.89 lower to 0.55 lower)	—	644 (7 RCTs)	⊕⊕⊕○ Moderate^a^
FOC (within 6‐week postpartum)	—	SMD 0.63 SD lower (0.91 lower to 0.35 lower)	—	397 (5 RCTs)	⊕○○○ Very low^a^, ^b^, ^c^
Pain catastrophising	—	SMD 0.36 SD lower (0.72 lower to 0.01 higher)	—	274 (3 RCTs)	⊕⊕⊕○ Moderate^c^
Mindfulness level (post‐intervention)	—	SMD 0.48 SD higher (0.31 higher to 0.66 higher)	—	682 (8 RCTs)	⊕○○○ Very low^a^, ^b^, ^d^
Mindfulness level (within 6‐week postpartum)	—	SMD 0.49 SD higher (0.27 higher to 0.71 higher)	—	321 (4 RCTs)	⊕⊕○○ Low^a^, ^c^
Pain intensity	—	SMD 1.22 SD lower (2.07 lower to 0.37 lower)	—	389 (5 RCTs)	⊕⊕○○ Low^b^, ^c^, ^d^
CS rate	275 per 1000	159 per 1000 (99 to 256)	RR 0.58 (0.36 to 0.93)	533 (6 RCTs)	⊕○○○ Very low^a^, ^b^, ^d^
EA rate	497 per 1000	383 per 1000 (288 to 507)	RR 0.77 (0.57 to 1.03)	329 (4 RCTs)	⊕○○○ Very low^b^, ^c^, ^d^
Total duration of labour	—	SMD 1.03 SD lower (1.34 lower to 0.72 higher)	—	180 (2 RCTs)	⊕⊕⊕○ Moderate^e^

Abbreviations: CI, confidence interval; CS, caesarean section; EA, epidural analgesia; FOC, fear of childbirth; RR, risk ratio; SMD, standardized mean difference.

^a^ Downgrade one level for the risk of bias due to high risk of bias studies.

^b^ Downgrade one level for the inconsistency due to the substantial heterogeneity.

^c^ Downgrade one level for the imprecision because the total sample size is less than 400.

^d^ Downgrade one level for the imprecision due to contradictory conclusions.

^e^ Downgrade one level for the imprecision because the total sample size is less than 200.

^f^ The risk in the intervention group (and its 95% confidence interval) is based on the assumed risk in the comparison group and the relative effect of the intervention (and its 95% CI).

In addition, the pooled results of the subgroup analysis indicated a smaller effect size post‐intervention and a larger effect size within 6 weeks post‐delivery in the studies that recruited only participants with a high level of FOC, without significant subgroup differences (Table S3, Figure S3a,b). Regarding the impacts of different control conditions, the pooled results of studies using usual care were larger than those studies employing active controls, although without significant subgroup differences at either immediate post‐intervention or 6‐week postpartum timepoints (Table S4, Figure S4a,b).

Furthermore, one study (Gökbulut et al. 2024) was excluded from meta‐analysis, as it reported only median and interquartile ranges, preventing us from evaluating whether the data were skewed, and it also reported significant baseline differences in FOC between two groups. Nevertheless, the study reported a lower FOC level post‐intervention (*p* < 0.001) in the intervention group compared with the control group.

#### 3.6.2 Pain Catastrophising

The meta‐analysis of three comparable studies that involved 274 participants revealed a nonsignificant change in pain catastrophising immediately post‐intervention in the intervention group, compared to the control group (SMD: −0.36; 95% CI −0.72, 0.01; *I* ^2^ = 50%; Table 2, Figure S1c), based on moderate certainty of evidence (Table 3).

#### 3.6.3 Mindfulness Level

For the immediate effect of the intervention, eight comparable studies measuring mindfulness levels included 682 participants. Very low certainty of evidence suggested that the intervention group reported significantly higher mindfulness levels immediately post‐intervention compared with the control group, with a medium effect size (SMD: 0.48, 95% CI 0.31, 0.66, *I* ^2^ = 19%) (Tables 2 and 3, Figure S1d). Regarding the effect within 6 weeks post‐delivery, pooled results of 303 participants from three studies revealed a significant increase in mindfulness level in the intervention group, compared to the control group, with a medium effect size (SMD: 0.50, 95% CI 0.27, 0.73, *I* ^2^ = 0%) (Table 2, Figure S1e), based on low certainty of evidence (Table 3).

Additionally, the subgroup analysis suggested no significant differences between studies adopting usual care and those with active controls at either immediate post‐intervention or 6‐week postpartum timepoints (Table S4, Figure S4c,d).

Moreover, one study (Beattie et al. 2017) measured the participants' mindfulness levels at 6 weeks post‐intervention (i.e., 6 weeks after 32–36 weeks of gestation). However, the study provided no information on whether the participants had delivered their baby. The results reported no significant difference between groups.

#### 3.6.4 Pain Intensity

Among the five studies that measured labour pain intensity, the severest pain intensity was selected for the meta‐analysis. Low certainty of evidence suggested that the participants in the intervention group experienced lower pain intensity than those in the control group, with a large effect size (SMD: −1.22; 95% CI −2.07, −0.37; *I* ^2^ = 93%; Tables 2 and 3, Figure S1f).

Furthermore, two studies were excluded from the meta‐analysis because they lacked standard deviation information (Duncan et al. 2017) or reported pain intensity as a categorical variable, without cut‐off value (Azh et al. 2021). Of the two studies, one revealed no significant difference in the average pain intensity during labour between two groups (Duncan et al. 2017), whereas the other study reported a significantly lower pain intensity in the intervention group at a cervical dilation of 4–6 cm, 7–8 cm, and 9–10 cm compared with the control group (Azh et al. 2021).

#### 3.6.5 Mode of Delivery

Six studies reported a CS rate of 533 participants. Based on very low certainty of evidence, the CS rate of the intervention group was significantly lower than that of the control group, with a medium effect size (RR: 0.58; 95% CI 0.36, 0.93; *I* ^2^ = 40%; Tables 2 and 3 Figure S1g). Besides, sensitivity analysis excluding one study rated as having a high risk of bias revealed consistent results (RR: 0.53, 95% CI 0.31, 0.91, *I* ^2^ = 48%) (Table S2, Figure S2e). Furthermore, the subgroup analysis of different control conditions revealed no statistically significant differences between subgroups (Table S4, Figure S4e).

No trial provided additional details, such as the reasons for or preference regarding CS.

#### 3.6.6 Pharmacological Pain Relief Method Use

Regarding pharmacological methods use, four studies that involved 329 participants reported the EA use rate. The pooled results suggested no significant difference in the EA rate between the two groups (RR: 0.77; 95% CI 0.57, 1.03; *I* ^2^ = 34%; Table 2, Figure S1h), based on very low certainty of evidence (Table 3). In addition, there was no statistically significant difference between studies adopting usual care and those with active controls (Table S4, Figure S4f).

Besides, two studies that reported the use of other pain relief methods, pethidine (Zhang et al. 2023) and narcotics (Duncan et al. 2017), reported nonsignificant results.

#### 3.6.7 Duration of Labour

The meta‐analysis of two comparable studies reporting total duration of labour contained 180 participants. Moderate certainty of evidence indicated a shorter total duration of labour in the intervention group, with a large effect size (SMD: −1.03; 95% CI −1.34, −0.72; *I* ^2^ = 0%; Tables 2 and 3, Figure S1i).

Of three studies reporting the duration of separated labour phases, two studies (Gao et al. 2024; Zhang et al. 2023) found no significant difference in the duration of the first, second, and third stages of labour between two groups, whereas the other study (Pan et al. 2020) reported a significantly shorter duration in the three stages for the intervention group, compared with the control group.

## 4 Discussion

This systematic review synthesized evidence from 15 trials on the effect of MBIs on FOC and related outcomes, and showed that MBIs may be effective in reducing FOC, labour pain intensity, the CS rate and the duration of labour, and enhancing mindfulness levels. However, this review detected no significant effect on pain catastrophising and the EA use rate.

Mindfulness is defined as awareness and acceptance of one's present‐moment experience (Creswell 2017). According to monitor and acceptance theory, attention monitoring and acceptance are two essential components of mindfulness practice (Lindsay and Creswell 2017). Specifically, though attention monitoring can enhance focus on present experiences and amplify positive and negative emotions, acceptance can weaken further emotional reactivity by facilitating early engagement in and disengagement from affective stimuli, which is a critical mechanism for mindfulness to promote health‐related outcomes (Lindsay and Creswell 2017). In mindfulness practice, participants are trained to observe their present‐moment experience by focusing on an object, such as breath, while avoiding distractions. To cultivate acceptance skills, participants are encouraged to welcome and approach every experience with curiosity and without judgment or immediate reaction, which can reduce emotional reactivity and promote stress‐related health outcomes.

This systematic review showed that MBIs could probably reduce FOC. This finding is consistent with that of a previous systematic review, despite slight differences in inclusion criteria regarding population, secondary outcomes, study design and publication languages (Abdolalipour et al. 2023). In this study, a relatively longer‐term effect was observed, which lasted up to 3 days or 1 week postpartum. However, the effect may diminish over time because two studies that measured FOC at 2–4 weeks or 6 weeks postpartum reported nonsignificant results (Duncan et al. 2017; Van der Meulen et al. 2023). The findings may be attributed to the short intervention duration (2.5 days) (Duncan et al. 2017) and the recruitment of only participants with a high level of FOC (Van der Meulen et al. 2023). Notably, Van der Meulen et al. (2023) argued that more intensive interventions, such as those that cover the entire perinatal period, including the postpartum phase, may be necessary, particularly for vulnerable pregnant women. Interestingly, the results of this review showed that the effect sizes of the population with a high level of FOC were smaller immediately post‐intervention but larger within 6 weeks postdelivery compared with those of the general population. This finding suggests that, for the individuals with a high level of FOC, considerable emphasis and intensive intervention during the antepartum period may be necessary to achieve the same effect.

This systematic review also observed the positive effect of MBIs on pain intensity reduction but without any impact on pain catastrophising. In coping with pain and pain catastrophising, mindfulness helps reframe pain by distinguishing it as a physical sensation rather than a source of suffering, which is often reinforced by negative cognitive interpretations (Hess 2018). This cognitive shift, along with perceiving childbirth as a productive rather than threatening experience, may explain the observed reductions in pain catastrophising and labour pain intensity (Whitburn et al. 2019). Therefore, MBIs aim to change individuals' relationship with subjective pain experience, promote acceptance and separate the sensation of pain from negative cognition, such as catastrophic thoughts (Hess 2018). However, in this systematic review, for pain catastrophising outcome, two of the three trials reported nonsignificant effects, possibly due to inadequate intervention intensity (Duncan et al. 2017) or the use of active control (Duncan et al. 2017; Zhang et al. 2023). Compared with another trial with significant results (Veringa‐Skiba, de Bruin, et al. 2022), which offered only two 90‐min consultation sessions on FOC, this trial provided childbirth courses of comparable length and quality to the intervention group (i.e., 8 weeks). The authors suggested that participants in the active control group received perinatal education, which contributed to a decrease of catastrophic thoughts and an improvement of overall well‐being (Zhang et al. 2023). Moreover, the intervention completion rates of these two studies were not satisfactory, which might also have an impact on the intervention effectiveness.

Regarding clinical outcomes, the results suggested uncertainty in the effect on the EA or CS rate because the certainty of evidence was very low. Previous literature demonstrated that MBIs can lead women to opt for a natural delivery without EA or elective CS, with mindfulness level serving as a mediator (Veringa‐Skiba, Ziemer, et al. 2022). The possible effect of MBIs on such outcomes may be partially attributed to the cultivation of acceptance skills and decrease of FOC. According to the theoretical model of avoidance and participation in pregnancy, birth and the postpartum period, women who tend to avoid a natural delivery prefer obstetric interventions such as a priori EA or elective CS (Veringa‐Skiba et al. 2016). As the mode of delivery might be affected by not only the participants' preferences but also medical factors, collecting reasons for CS would be valuable in understanding women's preference for natural delivery, which can reflect their acceptance. Similarly, information on the initial timing and total consumption of EA is important, as women with high levels of FOC are more likely to request EA before experiencing labour pain (Veringa‐Skiba et al. 2016). However, none of the studies included in this systematic review had reported this information, which restricted the further analysis of the effect of MBIs on the participants' participation in a natural delivery and their acceptance skills. Furthermore, the results revealed that MBIs could probably shorten the labour duration, which may be attributed to decreased sympathetic activity and stress after mindfulness practice (Wang, Zhang, et al. 2023). Excessive sympathetic activity is a key factor for incoordinate and decreased uterine contractions, which may lead to prolonged labour (Zagami et al. 2015).

In addition, the results indicated improvements in mindfulness levels; however, the certainty of evidence was very low or low. Given the mechanisms of the MBIs stated above, the enhancement of mindfulness levels is essential for improvements in the labour‐related outcomes. In this systematic review, the studies that reported a significant increase in the mindfulness level generally suggested improvements in other outcomes. Moreover, two studies that reported nonsignificant improvements in the mindfulness level provided a relatively short intervention, that is, 2.5 days (Duncan et al. 2017) and 2 days for an onsite course with self‐practice (Wang, Zhang, et al. 2023). Actually, the findings on the effective mindfulness practice duration are inconsistent across the current studies with different population and outcome measures. Nonetheless, researchers have suggested that consistent long‐term practice, albeit brief in daily duration, will have greater benefits than infrequent practice (Brintz et al. 2020).

### 4.1 Strengths and Limitations

This systematic review provided updated evidence on the effect of MBIs on FOC and filled the research gap on the effect of such interventions on mindfulness levels and clinical outcomes. Given the inconsistent and insufficient evidence, this systematic review is essential for clinical practice and further research.

Admittedly, this systematic review has some limitations. Firstly, the findings should be interpreted cautiously, as the overall methodological quality is low, and the certainty of evidence ranges from moderate to very low. In addition, the overall sample size remains relatively small. Regarding intervention implementation, overall adherence was not satisfactory, and only two studies reported measures to ensure fidelity. These limitations might have restricted accurate evaluation of intervention effectiveness, reduced replicability of the results, and caused challenges in attributing the findings to the intervention (Bradley et al. 2024). Moreover, while some studies reported mode of delivery and pharmacological pain relief methods use, only CS rate and pain relief method use rate were reported. However, delivery mode preferences, the initial timing and total consumption of pain relief method are also important to reflect FOC and women's participation in the labour process. Furthermore, only studies published in English and Chinese were included. This may introduce language bias and limit the generalisability of the results to broader populations.

### 4.2 Implications for Clinical Practice and Research

For clinical practice, integrating mindfulness practices into clinical care could enhance women's pregnancy and labour experiences while promoting overall well‐being. Despite these potential benefits, further research is needed to strengthen the evidence base. Future research should prioritize well‐designed RCTs with larger sample sizes. In addition, this systematic review explored the impacts of recruiting different populations (high FOC and general population) and use of different control conditions (usual care and active control) on intervention effectiveness. The nonsignificant subgroup results might be due to the limited number of studies and small sample size. Therefore, future studies should consider conducting subgroup analyses based on the baseline FOC level and select appropriate control conditions. Also, collecting more detailed information on mode of delivery, such as preference for or reasons for CS, and on pharmacological pain relief methods use, such as the preference, the initial timing and total consumption of EA, could be considered. Finally, using a theoretical model to guide the intervention development would help enhance the interpretability of the findings.

## 5 Conclusion

MBIs have been widely implemented among pregnant women. This systematic review provides evidence that MBIs have demonstrated an impact on reducing FOC, labour pain intensity, the CS rate, duration of labour, and improving mindfulness level, with medium to large effect sizes. These interventions might be an effective approach in obstetrics clinical practice to enhance the pregnancy and labour experiences among pregnant women. However, due to the very low to moderate certainty of evidence, these results should be interpreted with caution. Further RCTs with more rigorous methodologies and larger sample sizes are necessary to strengthen the evidence.

## Funding

The authors have nothing to report.

## Conflicts of Interest

The authors declare no conflicts of interest.

## Supporting information


**Data S1:** wvn70101‐sup‐0001‐Supinfo.docx.

## Data Availability

The data that support the findings of this study are available from the corresponding author upon reasonable request.
