# Supplementary material for: Effectiveness of Mindfulness‐Based Interventions for Fear of Childbirth Among Pregnant Women Planned for Normal Birth: A Systematic Review and Meta‐Analysis
Source: Worldviews Evid Based Nurs. 2026 Jan 19;23(1):e70101. doi: 10.1111/wvn.70101 (PMC12816443; doi:10.1111/wvn.70101)

**Supplementary Table 1**

Search record in different databases

| **Medline (via Pubmed)** | | **Results** |
| --- | --- | --- |
| #1 | "Parturition"[MeSH Terms] OR "delivery, obstetric"[MeSH Terms] OR "labor, obstetric"[MeSH Terms] OR "parturi*"[Title/Abstract] OR "delivery"[Title/Abstract] OR "labor"[Title/Abstract] OR "labour"[Title/Abstract] OR "birth"[Title/Abstract] OR "childbirth"[Title/Abstract] OR "maternal"[Title/Abstract] OR "intrapartum"[Title/Abstract] OR "nullipar*"[Title/Abstract] OR "multipar*"[Title/Abstract] OR "primipar*"[Title/Abstract] OR "primigravida"[Title/Abstract] | 1,376,573 |
| #2 | "Mindfulness"[MeSH Terms] OR "Meditation"[MeSH Terms] OR "Mindful*"[Title/Abstract] OR "Meditation"[Title/Abstract] | 25,478 |
| #3 | "Fear"[MeSH Terms] OR "Fear"[Title/Abstract] OR "Tocophobia"[Title/Abstract] OR "Tokophobia"[Title/Abstract] OR "parturiphobia"[Title/Abstract] | 108,956 |
| #4 | "Labor Pain"[MeSH Terms] OR "Pain Management"[MeSH Terms] OR "Pain"[MeSH Terms] OR "Pain"[Title/Abstract] | 1,012,524 |
| #5 | "Cesarean Section"[MeSH Terms] OR "cesarean"[Title/Abstract] OR "cesarean"[Title/Abstract] | 79,367 |
| #6 | #3 OR #4 OR #5 | 1,183,151 |
| #7 | "Randomized Controlled Trial"[Publication Type] OR "random*"[Title/Abstract] OR "RCT"[Title/Abstract] OR "intervention"[Title/Abstract] | 2,374,696 |
| #8 | #1 AND #2 AND #6 AND #7 | 135 |
|  |  |  |
| **Embase (via Ovid)** | | **Results** |
| 1 | exp birth/ | 38,550 |
| 2 | delivery.mp. or exp obstetric delivery/ | 1,246,878 |
| 3 | labor.mp. or exp "Labor (Childbirth)"/ | 212,289 |
| 4 | (parturi* or delivery or labor or labour or birth or childbirth or maternal or intrapartum or nullipar* or multipar* or primipar* or primigravida).mp. | 2,188,920 |
| 5 | 1 or 2 or 3 or 4 | 2,240,590 |
| 6 | Mindfulness.mp. or exp mindfulness/ | 23,496 |
| 7 | exp meditation/ or Meditation.mp. | 14,768 |
| 8 | (mindful* or Meditation).mp. | 38,329 |
| 9 | 6 or 7 or 8 | 38,329 |
| 10 | fear/ or "fear of childbirth"/ or fear.mp. | 154,310 |
| 11 | Tocophobia.mp. | 58 |
| 12 | Tokophobia.mp. | 78 |
| 13 | parturiphobia.mp. | 3 |
| 14 | 10 or 11 or 12 or 13 | 154,333 |
| 15 | exp pain/ or exp labor pain/ | 1,863,320 |
| 16 | pain.mp. | 1,765,531 |
| 17 | 15 or 16 | 2,233,151 |
| 18 | Cesarean Section.mp. or exp cesarean section/ | 150,805 |
| 19 | cesarean.mp. | 157,337 |
| 20 | caesarean.mp. | 46,999 |
| 21 | 18 or 19 or 20 | 164,569 |
| 22 | 14 or 17 or 21 | 2,510,163 |
| 23 | randomized controlled trial.mp. or exp randomized controlled trial/ | 1,157,501 |
| 24 | (random* or RCT or intervention).mp. | 3,532,108 |
| 25 | 23 or 24 | 3,533,265 |
| 26 | 5 and 9 and 22 and 25 | 273 |
|  |  |  |
| **CENTRAL** | | **Results** |
| #1 | MeSH descriptor: [Parturition] explode all trees | 917 |
| #2 | MeSH descriptor: [Labor, Obstetric] explode all trees | 3,269 |
| #3 | MeSH descriptor: [Delivery, Obstetric] explode all trees | 7,674 |
| #4 | (parturi* or delivery or labor or labour or birth or childbirth or maternal or intrapartum or nullipar* or multipar* or primipar* or primigravida):ti,ab,kw | 121,945 |
| #5 | #1 or #2 or #3 or #4 | 123,189 |
| #6 | MeSH descriptor: [Mindfulness] explode all trees | 2,464 |
| #7 | MeSH descriptor: [Meditation] explode all trees | 1,132 |
| #8 | (mindful* or meditation):ti,ab,kw | 13,561 |
| #9 | #6 or #7 or #8 | 13,583 |
| #10 | MeSH descriptor: [Fear] explode all trees | 2,444 |
| #11 | (Fear or Tocophobia or Tokophobia or Parturiphobia):ti,ab,kw | 14,359 |
| #12 | #10 or #11 | 14,511 |
| #13 | MeSH descriptor: [Labor Pain] explode all trees | 593 |
| #14 | MeSH descriptor: [Pain] explode all trees | 73,908 |
| #15 | (pain):ti,ab,kw | 264,271 |
| #16 | #13 or #14 or #15 | 272,181 |
| #17 | MeSH descriptor: [Cesarean Section] explode all trees | 4,624 |
| #18 | (cesarean or caesarean):ti,ab,kw | 18,608 |
| #19 | #17 or #18 | 18,608 |
| #20 | #12 or #16 or #19 | 296,124 |
| #21 | MeSH descriptor: [Randomized Controlled Trial] explode all trees | 37 |
| #22 | (random* or RCT or intervention):ti,ab,kw | 1,629,606 |
| #23 | #13 or #14 | 1,629,606 |
| #24 | #5 and #8 and #12 and #15 | 244 |
|  |  |  |
| **CINAHL** | | **Results** |
| S1 | MH parturition OR MH delivery, obstetric OR MH labor, obstetric OR SU ( parturi* or delivery or labor or labour or birth or childbirth or maternal or intrapartum or nullipar* or multipar* or primipar* or primigravida ) | 104,568 |
| S2 | MH mindfulness OR MH meditation OR SU mindful* OR SU meditation | 7,679 |
| S3 | SU ( Fear or Tocophobia or Tokophobia or Parturiphobia ) OR MH fear | 9,209 |
| S4 | MH labor pain OR MH pain OR MH pain management OR SU pain | 69,445 |
| S5 | MH Cesarean Section OR SU cesarean OR SU caesarean | 6,053 |
| S6 | S3 OR S4 OR S5 | 83,443 |
| S7 | MH randomized controlled trials OR SU ( random* OR RCT OR intervention ) | 104,628 |
| S8 | S1 AND S2 AND S6 AND S7 | 7 |
|  |  |  |
| **PsycINFO (via Ovid)** | | **Results** |
| 1 | exp birth/ | 26,547 |
| 2 | delivery.mp. or exp obstetric delivery/ | 99,683 |
| 3 | labor.mp. or exp "Labor (Childbirth)"/ | 46,835 |
| 4 | (parturi* or delivery or labor or labour or birth or childbirth or maternal or intrapartum or nullipar* or multipar* or primipar* or primigravida).mp. | 278,398 |
| 5 | 1 or 2 or 3 or 4 | 281,654 |
| 6 | Mindfulness.mp. or exp mindfulness/ | 23,342 |
| 7 | exp meditation/ or Meditation.mp. | 11,229 |
| 8 | (mindful* or Meditation).mp. | 33,350 |
| 9 | 6 or 7 or 8 | 33,350 |
| 10 | fear/ or "fear of childbirth"/ or fear.mp. | 91,683 |
| 11 | Tocophobia.mp. | 15 |
| 12 | Tokophobia.mp. | 39 |
| 13 | parturiphobia.mp. | 0 |
| 14 | 10 or 11 or 12 or 13 | 91,687 |
| 15 | exp pain/ or exp labor pain/ | 70,887 |
| 16 | pain.mp. | 124,427 |
| 17 | 15 or 16 | 135,768 |
| 18 | Cesarean Section.mp. or exp cesarean section/ | 1,340 |
| 19 | cesarean.mp. | 1,937 |
| 20 | caesarean.mp. | 1,034 |
| 21 | 18 or 19 or 20 | 2,541 |
| 22 | 14 or 17 or 21 | 223,838 |
| 23 | randomized controlled trial.mp. or exp randomized controlled trial/ | 28,742 |
| 24 | (random* or RCT or intervention).mp. | 573,135 |
| 25 | 23 or 24 | 573,135 |
| 26 | 5 and 9 and 22 and 25 | 78 |
|  |  |  |
| **Dissertations & Theses (via ProQuest)** | | **Results** |
| S1 | mainsubject.Exact("childbirth & labor") OR title(parturi* or delivery or labor or labour or birth or childbirth or maternal or intrapartum or nullipar* or multipar* or primipar* or primigravida) | 47,103 |
| S2 | mainsubject.Exact("mindfulness" OR "meditation") OR title(mindful* or Meditation) | 5,428 |
| S3 | mainsubject.Exact("fear") OR title(Fear or Tocophobia or Tokophobia or Parturiphobia) | 4,028 |
| S4 | mainsubject.Exact("pain") OR title(pain) | 15,101 |
| S5 | mainsubject.Exact("cesarean section") OR title(cesarean) OR title(caesarean) | 861 |
| S6 | [S3] OR [S4] OR [S5] | 19,797 |
| S7 | mainsubject.Exact("clinical trials") OR title(Randomized Controlled Trial, Randomised Controlled Trial, random* OR RCT OR intervention) | 33,753 |
| S8 | [S1] AND [S2] AND [S6] AND [S7] | 0 |
|  |  |  |
| **CNKI** | | **Results** |
| 1 | (SU=分娩) AND ( SU=恐惧 OR SU=害怕 OR SU=惊恐 OR SU=疼痛 OR SU=无痛 OR SU=镇痛 OR SU=产痛 OR SU=剖宫产) AND (SU=正念 OR SU=冥想 OR SU=静观) AND (SU=随机对照试验 or SU=临床试验 OR SU=随机 OR SU=干预) | 31 |
|  |  |  |
| **WanFang** | | **Results** |
| 1 | 题名或关键词: ((分娩) and (恐惧 or 害怕 or 惊恐 or 疼痛 or 无痛 or 镇痛 or 产痛 or 剖宫产) and (正念 or 冥想 or 静观) and (随机对照试验 or 临床试验 or 随机 or 干预)) | 9 |
|  |  |  |
| **VIP** | | **Results** |
| 1 | M= 分娩 and M=(恐惧 or 害怕 or 惊恐 or 疼痛 or 无痛 or 镇痛 or 产痛 or 剖宫产) and M=(正念 or 冥想 or 静观) and M=(随机对照试验 or 临床试验 or 随机 or 干预) | 61 |
|  |  |  |
| **CBM (via SinoMed)** | | **Results** |
| 1 | ("分娩"[常用字段:智能]) OR ("分娩"[不加权:扩展]) | 216,783 |
| 2 | ("正念"[不加权:扩展]) OR ("正念"[常用字段:智能] OR "冥想"[常用字段:智能] AND "静观"[常用字段:智能]) | 3,076 |
| 3 | ("恐惧"[常用字段:智能] OR "害怕"[常用字段:智能] OR "惊恐"[常用字段:智能]) OR ("恐惧"[不加权:扩展]) | 35,442 |
| 4 | ("疼痛"[常用字段:智能] OR "无痛"[常用字段:智能] OR "镇痛"[常用字段:智能] OR "产痛"[常用字段:智能]) OR ("疼痛"[不加权:扩展] OR "分娩疼痛"[不加权:扩展]) | 1,620,910 |
| 5 | ("剖宫产"[常用字段:智能]) OR ("剖宫产术"[不加权:扩展]) | 190,336 |
| 6 | 3 or 4 or 5 | 1,923,823 |
| 7 | ("随机对照试验"[不加权:扩展]) OR ("随机对照试验"[常用字段:智能] OR "临床试验"[常用字段:智能] OR "随机"[常用字段:智能] OR "干预"[常用字段:智能]) | 2,413,034 |
| 8 | 1 and 2 and 6 and 7 | 33 |

**Supplementary Table 2**

Sensitivity analysis excluding high risk of bias studies

| **Outcomes** | **No. of study** | **Sample size** | **SMD/RR (95% CI)** | ***P*** | ***I^2^*, %** |
| --- | --- | --- | --- | --- | --- |
| FOC (post-intervention) | 6 | 538 | -0.72 (-0.93, -0.52) | <0.001^a^ | 24 |
| ITT analysis | 5 | 455 | -0.71 (-0.96, -0.46) | <0.001 ^a^ | 37 |
| PP analysis | 1 | 83 | -0.81 (-1.26, -0.37) | <0.001 ^a^ | - |
| FOC (within 6-week postpartum) | 4 | 291 | -0.53 (-0.77, -0.30) | <0.001 ^a^ | 0 |
| ITT analysis | 3 | 208 | -0.56 (-0.84, -0.28) | <0.001 ^a^ | 23 |
| PP analysis | 1 | 83 | -0.46 (-0.89, -0.02) | 0.040 ^a^ | - |
| Mindfulness level (post-intervention) | 6 | 556 | 0.53 (0.32, 0.73) | <0.001 ^a^ | 27 |
| ITT analysis | 4 | 378 | 0.52 (0.21, 0.84) | 0.001 ^a^ | 53 |
| PP analysis | 2 | 178 | 0.48 (0.19, 0.78) | 0.001 ^a^ | 0 |
| Mindfulness level (within 6-week postpartum) | 2 | 197 | 0.58 (0.29, 0.86) | <0.001 ^a^ | 0 |
| ITT analysis | 1 | 114 | 0.54 (0.17, 0.92) | 0.004 ^a^ | - |
| PP analysis | 1 | 83 | 0.62 (0.18, 1.06) | 0.006 ^a^ | - |
| CS rate | 5 | 514 | 0.53 (0.31, 0.91) | 0.020 ^a^ | 48 |

**Abbreviations:** CS: Caesarean section; FOC: fear of childbirth; ITT: intention-to-treat; PP: per-protocol

^a^ *P*<0.05

**Supplementary Table 3**

Subgroup analysis between studies recruiting high fear of childbirth (FOC) women and other studies

| **Outcomes** | **No. of study** | **Sample size** | **SMD (95% CI)** | ***P*** | ***I^2^*, %** |
| --- | --- | --- | --- | --- | --- |
| FOC (post-intervention) | 7 | 644 | -0.72 (-0.88, -0.56) | <0.001 ^a^ | 9 |
| High FOC | 3 | 352 | -0.61 (-0.83, -0.40) | <0.001 ^a^ | 0 |
| All levels of FOC | 4 | 292 | -0.85 (-1.09, -0.61) | <0.001 ^a^ | 0 |
| FOC (within 6-week postpartum) | 5 | 397 | -0.63 (-0.91, -0.35) | <0.001 ^a^ | 46 |
| High FOC | 3 | 285 | -0.75 (-1.12, -0.37) | <0.001 ^a^ | 56 |
| All levels of FOC | 2 | 112 | -0.40 (-0.78, -0.03) | 0.030 ^a^ | 0 |

**Abbreviations:** FOC: fear of childbirth

^a^ *P*<0.05

**Supplementary Table 4**

Subgroup analysis between studies applying usual care and active control

| **Outcomes** | **No. of study** | **Sample size** | **SMD (95% CI)** | ***P*** | ***I^2^*, %** |
| --- | --- | --- | --- | --- | --- |
| FOC (post-intervention) | 7 | 644 | -0.72 (-0.88, -0.56) | <0.001 ^a^ | 9 |
| Usual care | 4 | 400 | -0.83 (-1.03, -0.62) | <0.001 ^a^ | 0 |
| Active control | 3 | 244 | -0.54 (-0.80, -0.28) | <0.001 ^a^ | 7 |
| FOC (within 6-week postpartum) | 5 | 397 | -0.63 (-0.91, -0.35) | <0.001 ^a^ | 46 |
| Usual care | 3 | 285 | -0.75 (-1.12, -0.37) | <0.001 ^a^ | 56 |
| Active control | 2 | 112 | -0.40 (-0.78, -0.03) | 0.030 ^a^ | 0 |
| Mindfulness level (post-intervention) | 8 | 682 | 0.48 (0.31, 0.66) | <0.001 ^a^ | 19 |
| Usual care | 3 | 320 | 0.57 (0.26, 0.87) | <0.001 ^a^ | 45 |
| Active control | 5 | 362 | 0.42 (0.20, 0.64) | <0.001 ^a^ | 5 |
| Mindfulness level (within 6-week postpartum) | 3 | 303 | 0.50 (0.27, 0.73) | <0.001 ^a^ | 0 |
| Usual care | 2 | 220 | 0.45 (0.18, 0.72) | <0.001 ^a^ | 0 |
| Active control | 1 | 83 | 0.62 (0.18, 1.06) | 0.006 ^a^ | - |
| CS rate | 6 | 533 | 0.58 (0.36, 0.93) | 0.020 ^a^ | 40 |
| Usual care | 3 | 265 | 0.53 (0.26, 1.06) | 0.070 | 55 |
| Active control | 3 | 268 | 0.60 (0.23, 1.52) | 0.280 | 47 |
| EA rate | 4 | 329 | 0.77 (0.57, 1.03) | 0.070 | 34 |
| Usual care | 1 | 85 | 0.60 (0.38, 0.95) | 0.030 ^a^ | - |
| Active control | 3 | 244 | 0.84 (0.59, 1.19) | 0.320 | 34 |

**Abbreviations:** CS: Caesarean section; EA: Epidural analgesia; FOC: fear of childbirth

^a^ *P*<0.05

**Supplementary Figure 1**

Forest plot of main analysis

**1a. FOC (post-intervention)**


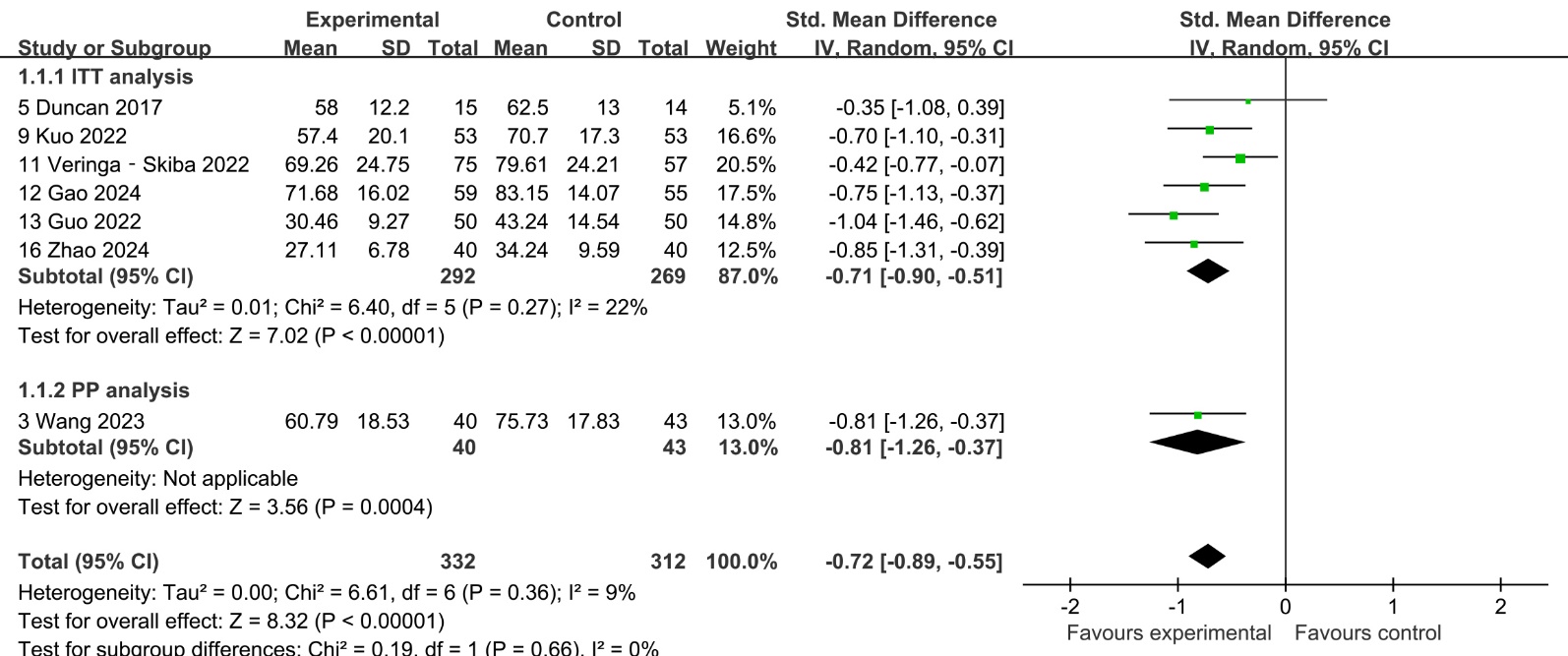


**1b. FOC (within 6-week postpartum)**


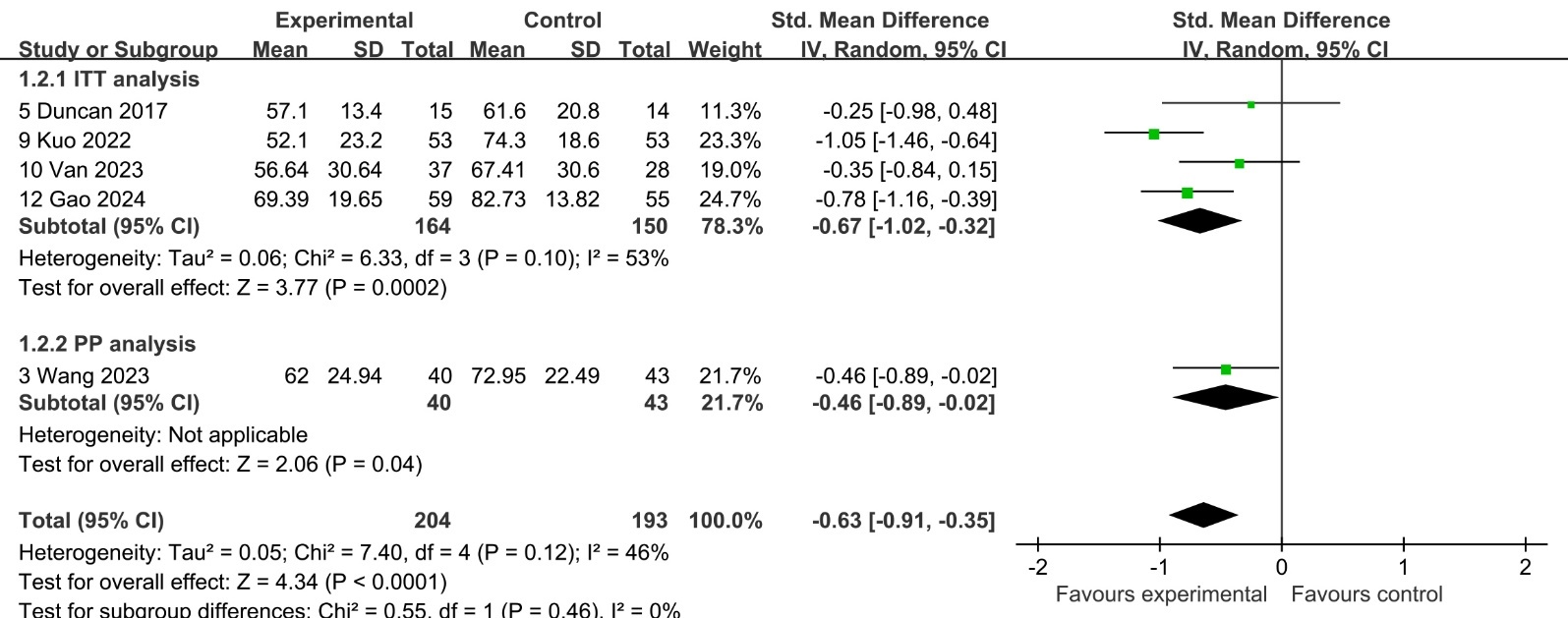


**1c. Pain catastrophising**


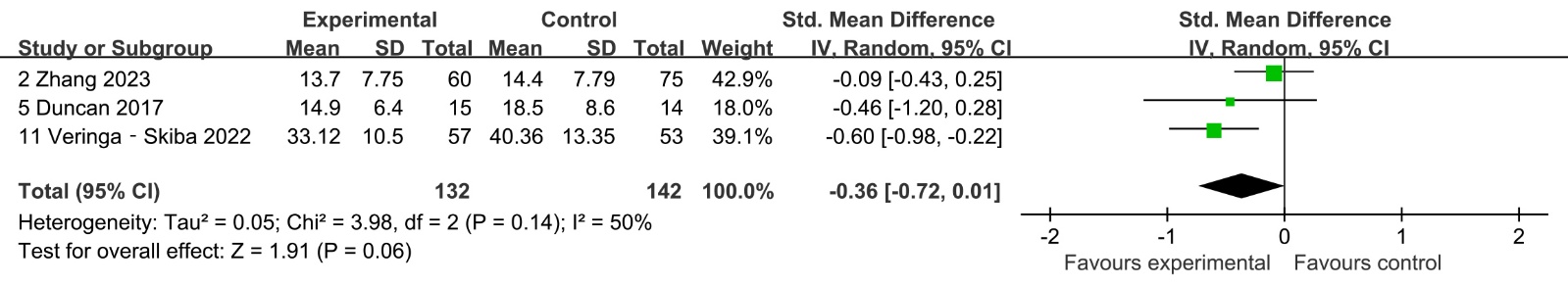


**1d. Mindfulness level (post-intervention)**


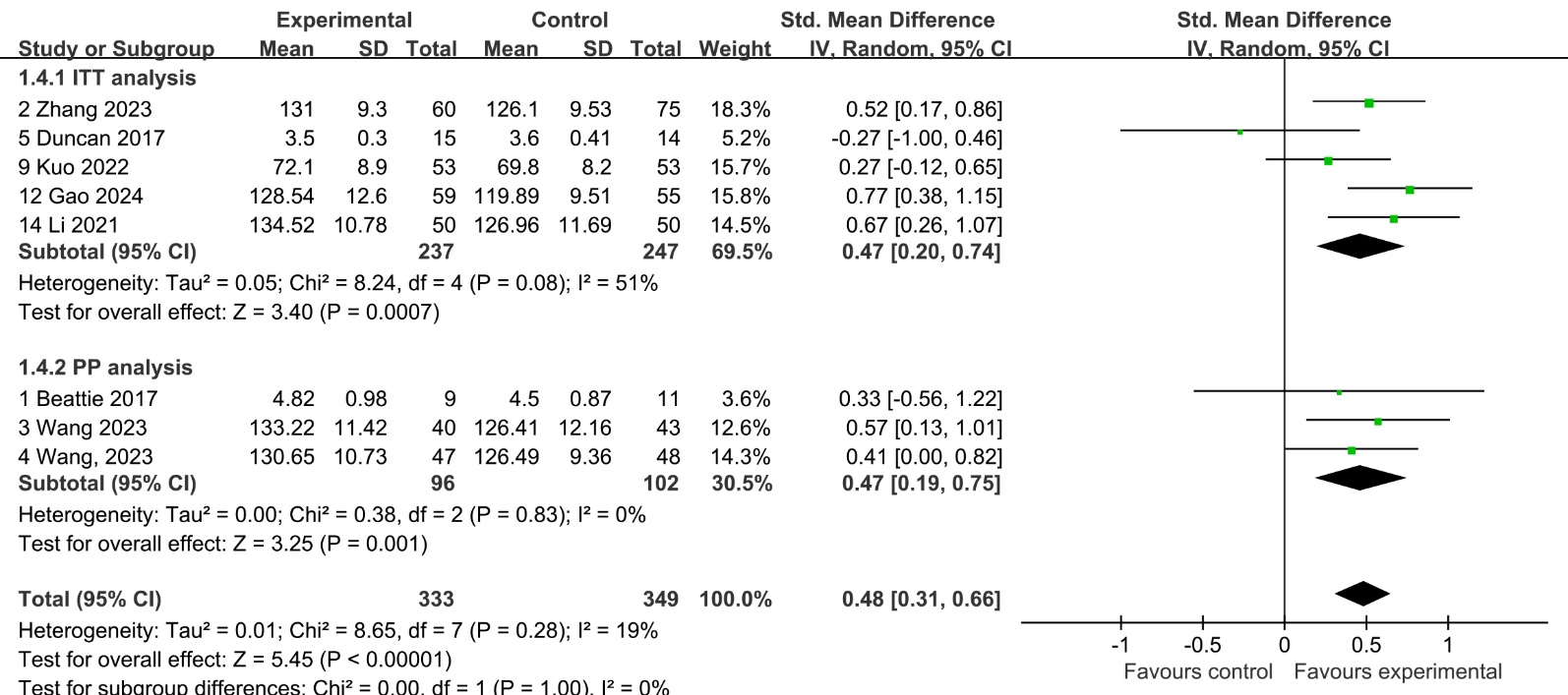


**1e. Mindfulness level (within 6-week postpartum)**


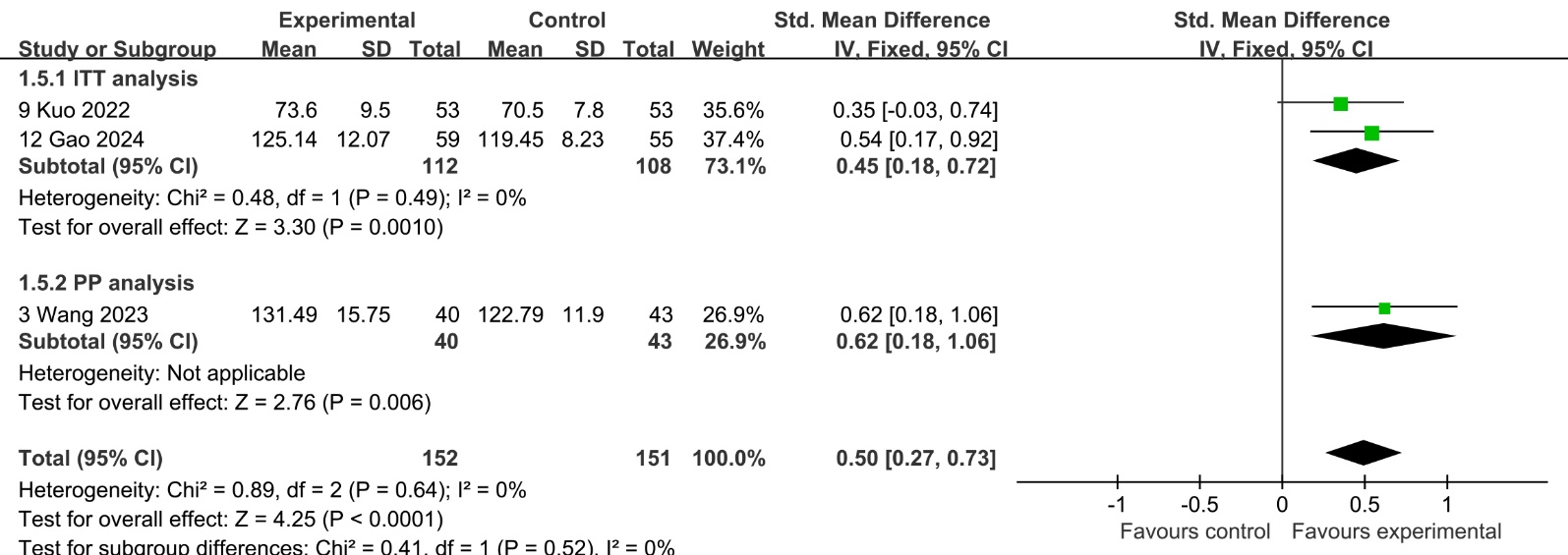


**1f. Pain intensity**


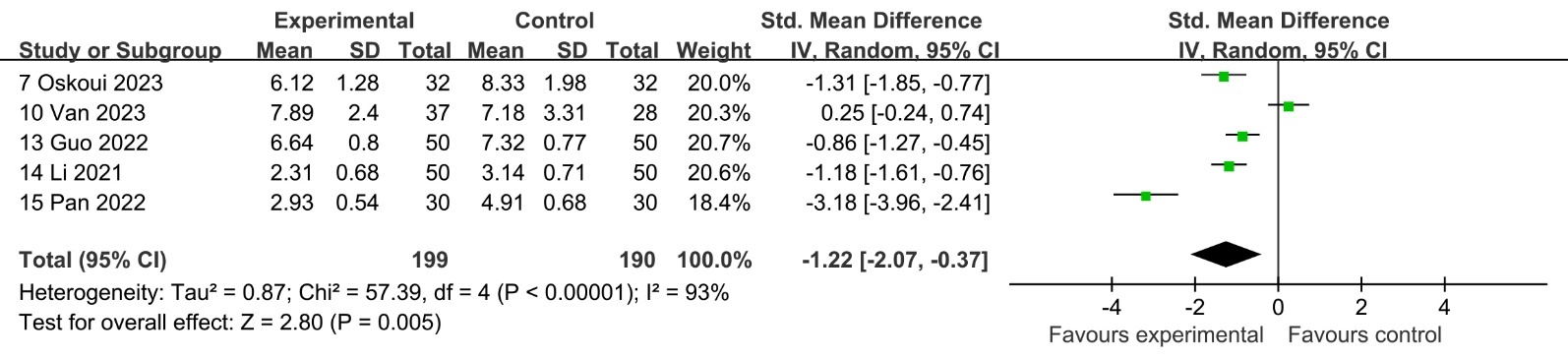


**1g. Caesarean section rate**


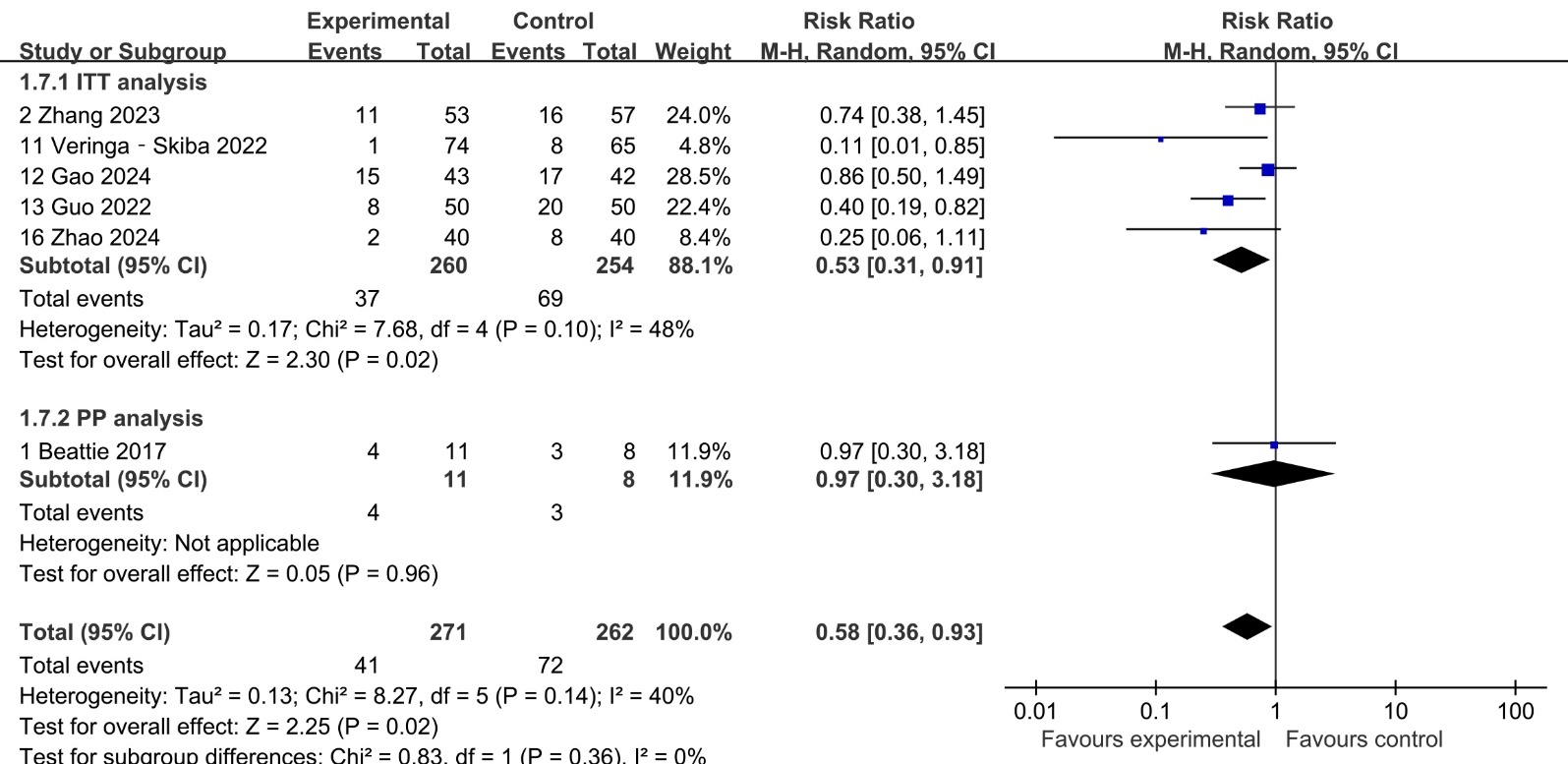


**1h. Epidural analgesia use rate**


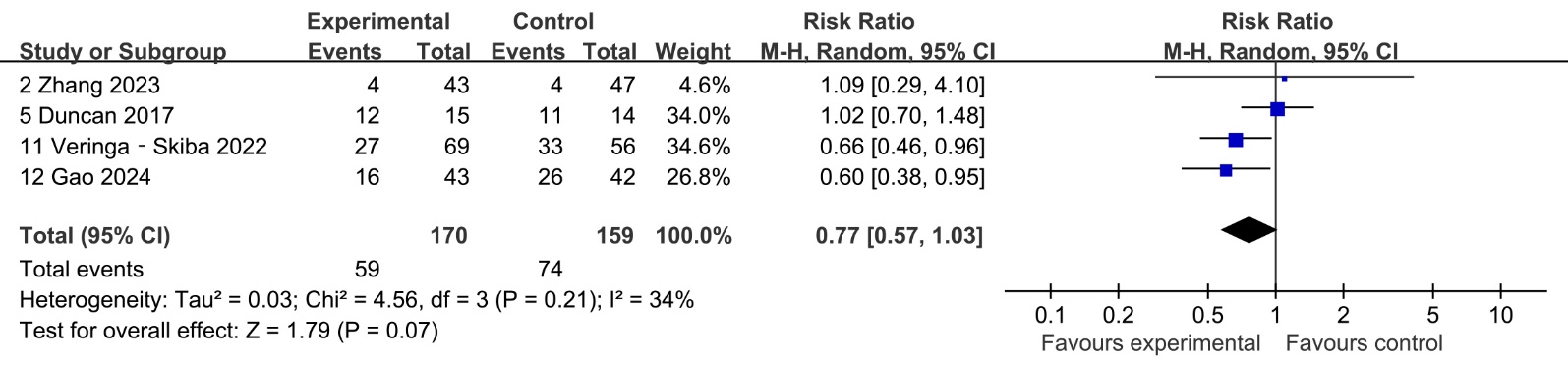


**1i. Total duration of labour**


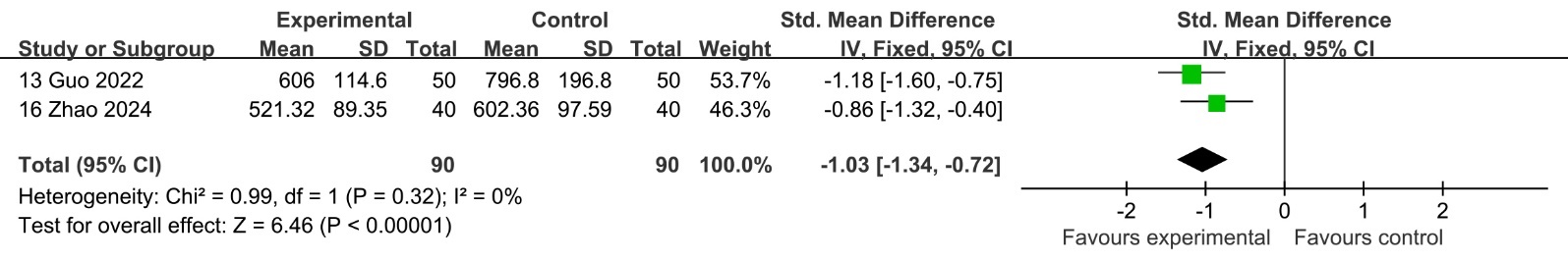


**Supplementary Figure 2**

Forest plot of sensitivity analysis excluding high risk of bias studies

**2a. Sensitivity analysis excluding high risk of bias studies: FOC (post-intervention)**


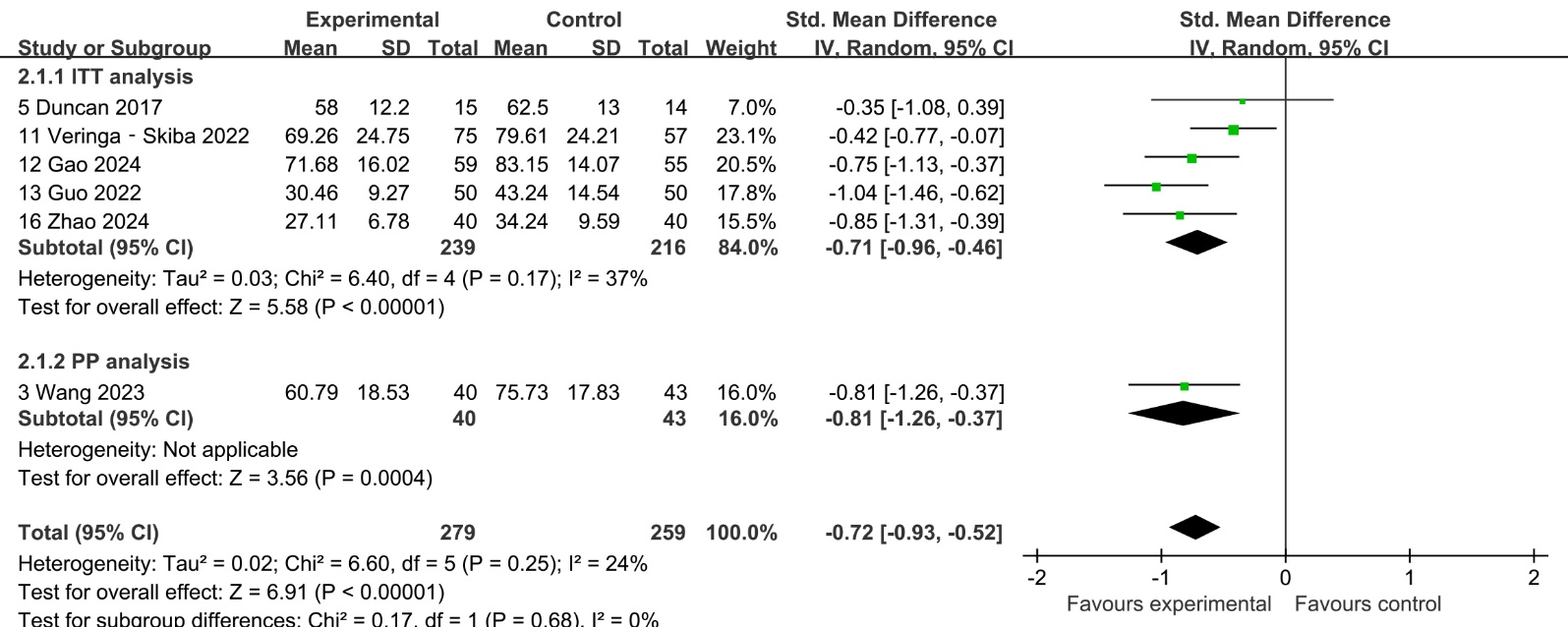


**2b. Sensitivity analysis excluding high risk of bias studies: FOC (within 6-week postpartum)**


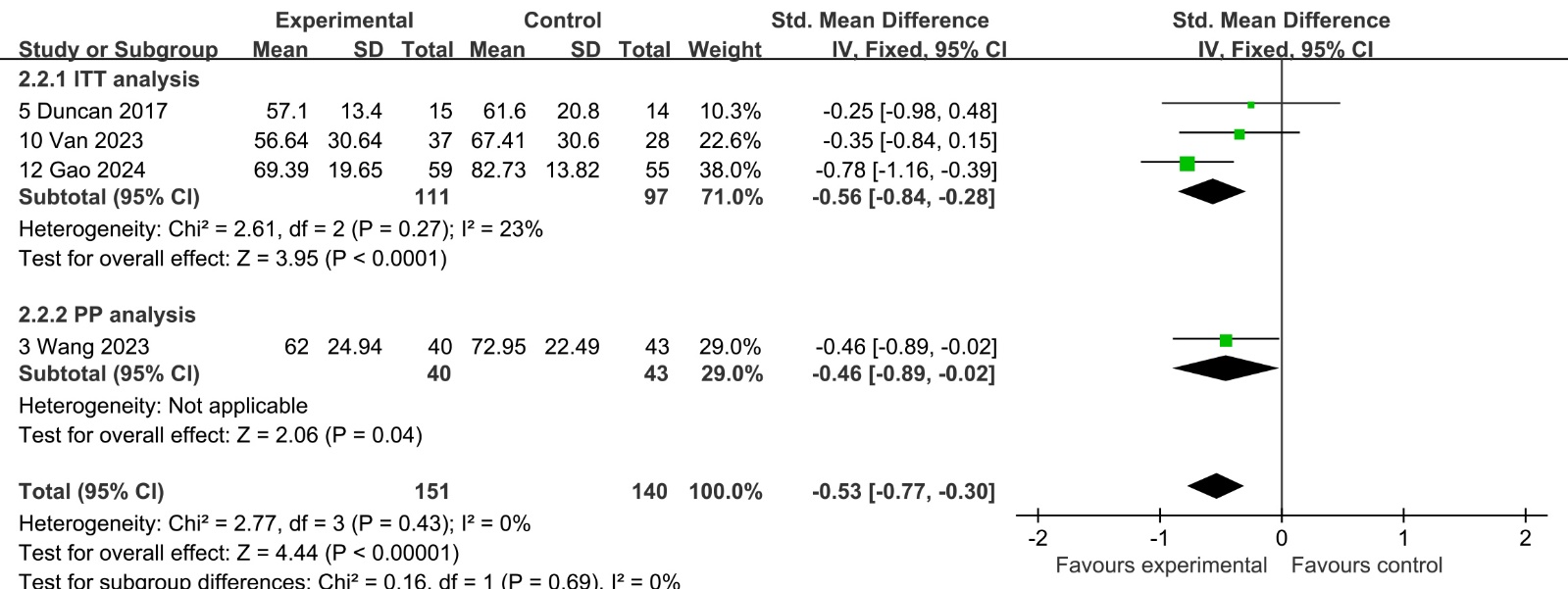


**2c. Sensitivity analysis excluding high risk of bias studies: mindfulness level (post-intervention)**


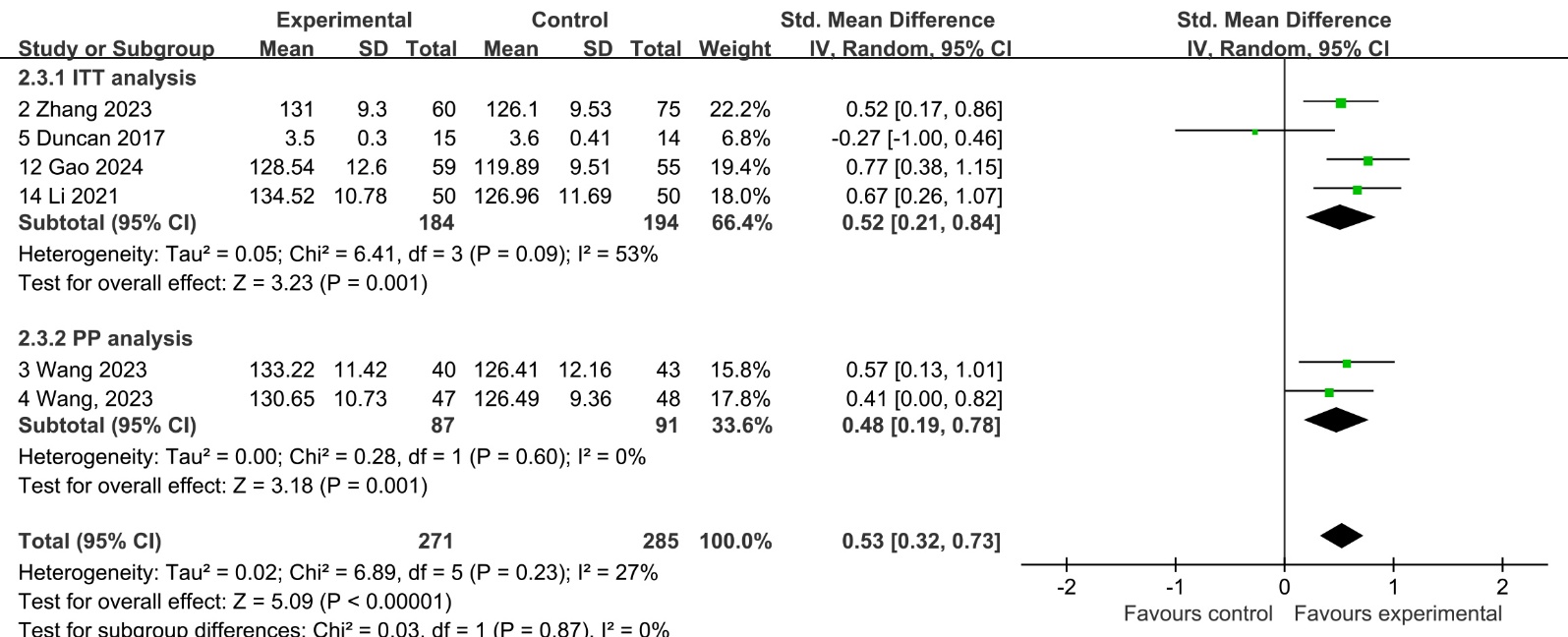


**2d. Sensitivity analysis excluding high risk of bias studies: mindfulness level (within 6-week postpartum)**


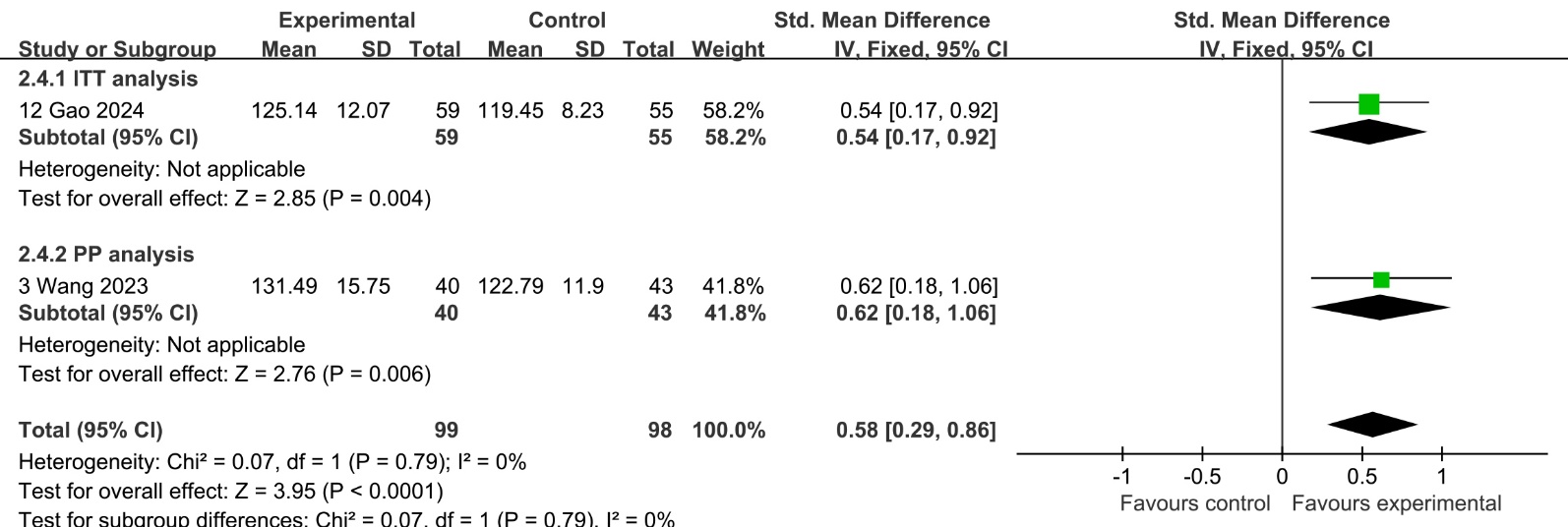


**2e. Sensitivity analysis excluding high risk of bias studies: caesarean section rate**


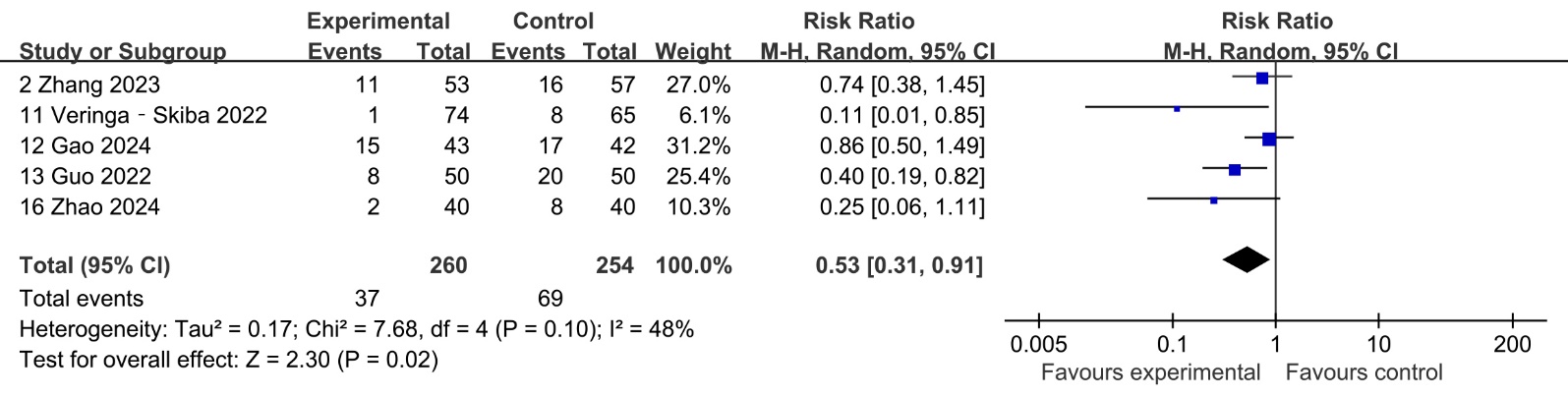


**Supplementary Figure 3**

Forest plot of subgroup analysis between studies recruiting high FOC women and other studies

**3a. Subgroup analysis between studies recruiting high FOC women and other studies: FOC (post-intervention)**


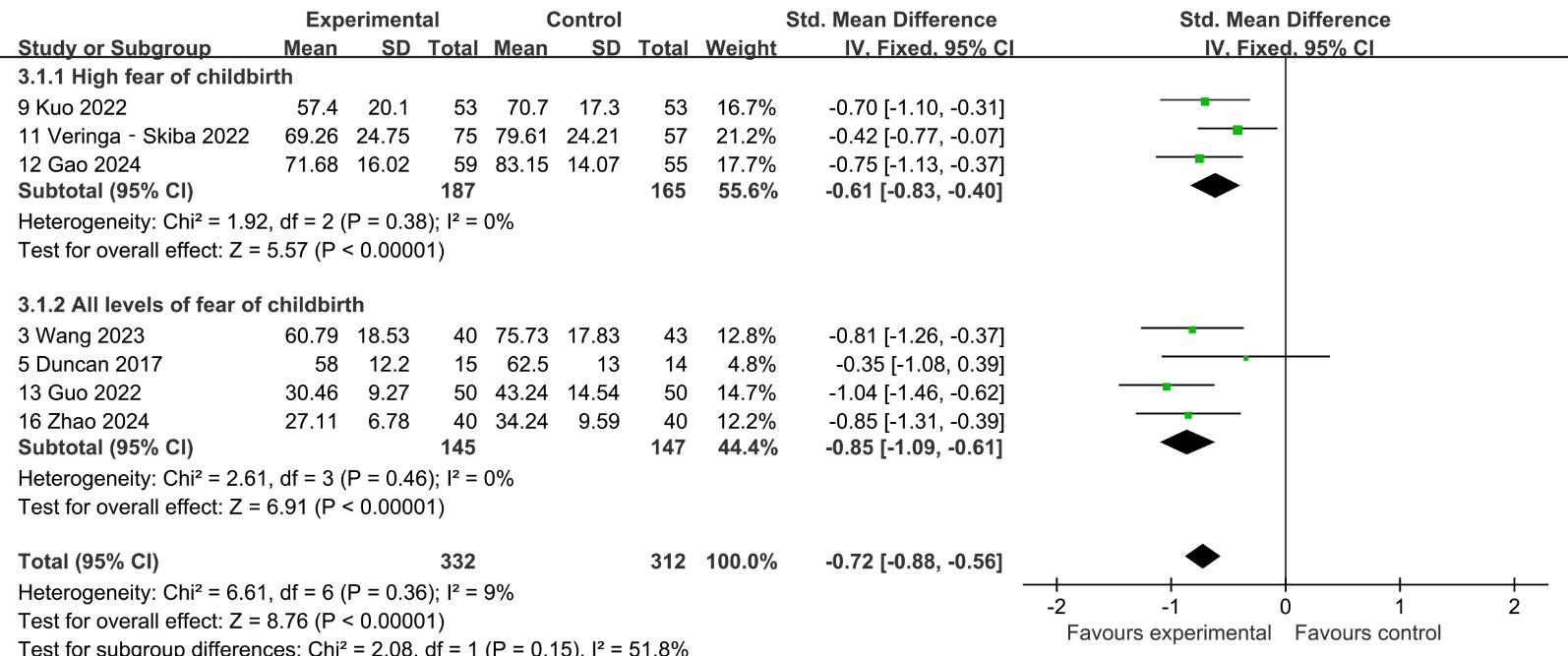


**3b. Subgroup analysis between studies recruiting high FOC women and other studies: FOC (within 6-week postpartum)**


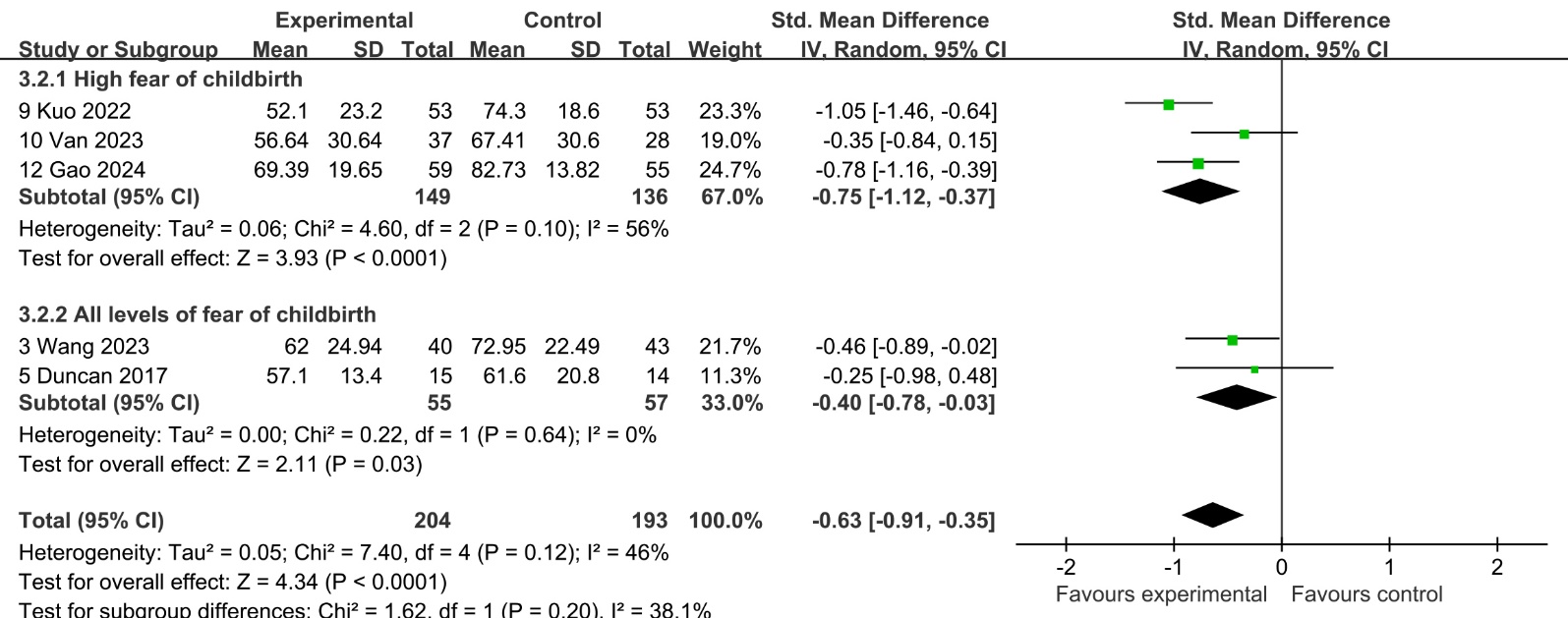


**Supplementary Figure 4**

Forest plot of subgroup analysis between studies applying usual care and active control

**4a. Subgroup analysis between studies applying usual care and active control: FOC (post-intervention)**


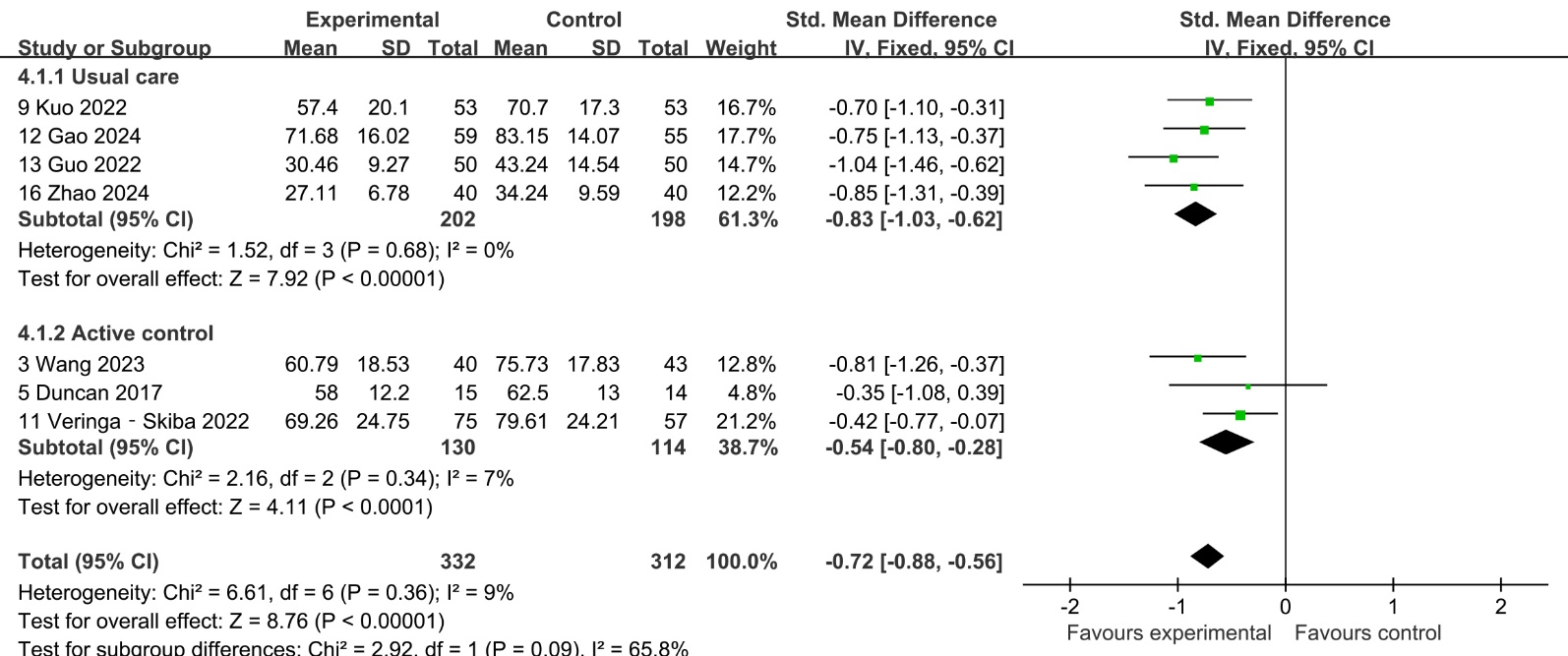


**4b. Subgroup analysis between studies applying usual care and active control: FOC (within 6-week postpartum)**


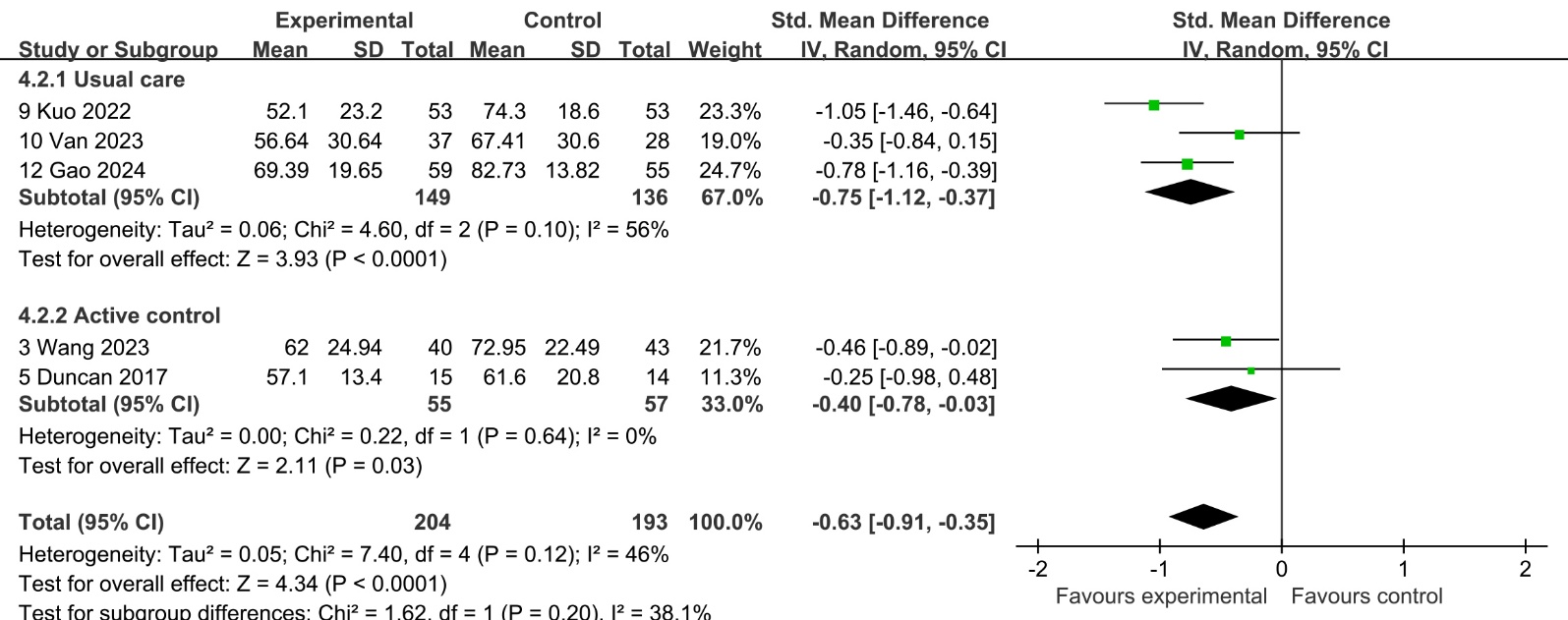


**4c. Subgroup analysis between studies applying usual care and active control: Mindfulness level (post-intervention)**


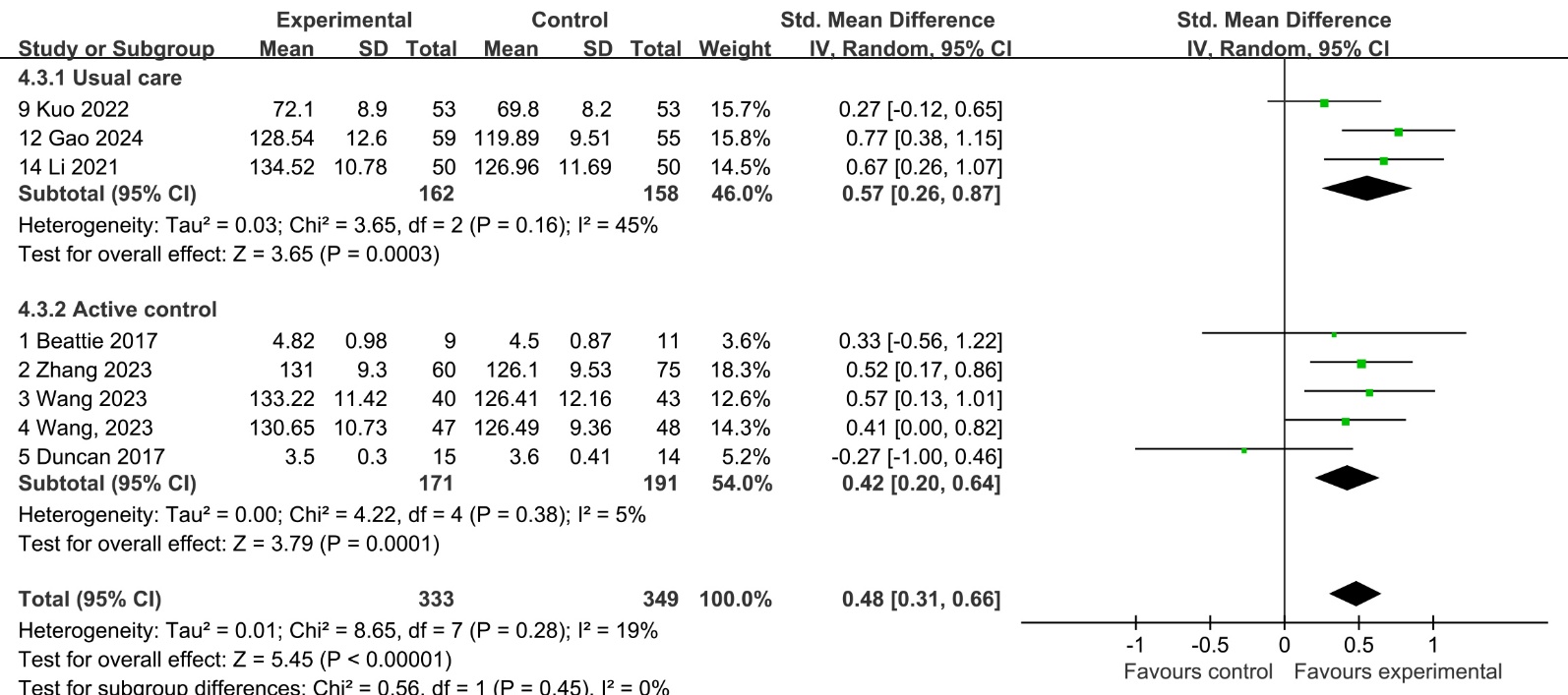


**4d. Subgroup analysis between studies applying usual care and active control: Mindfulness level (within 6-week postpartum)**


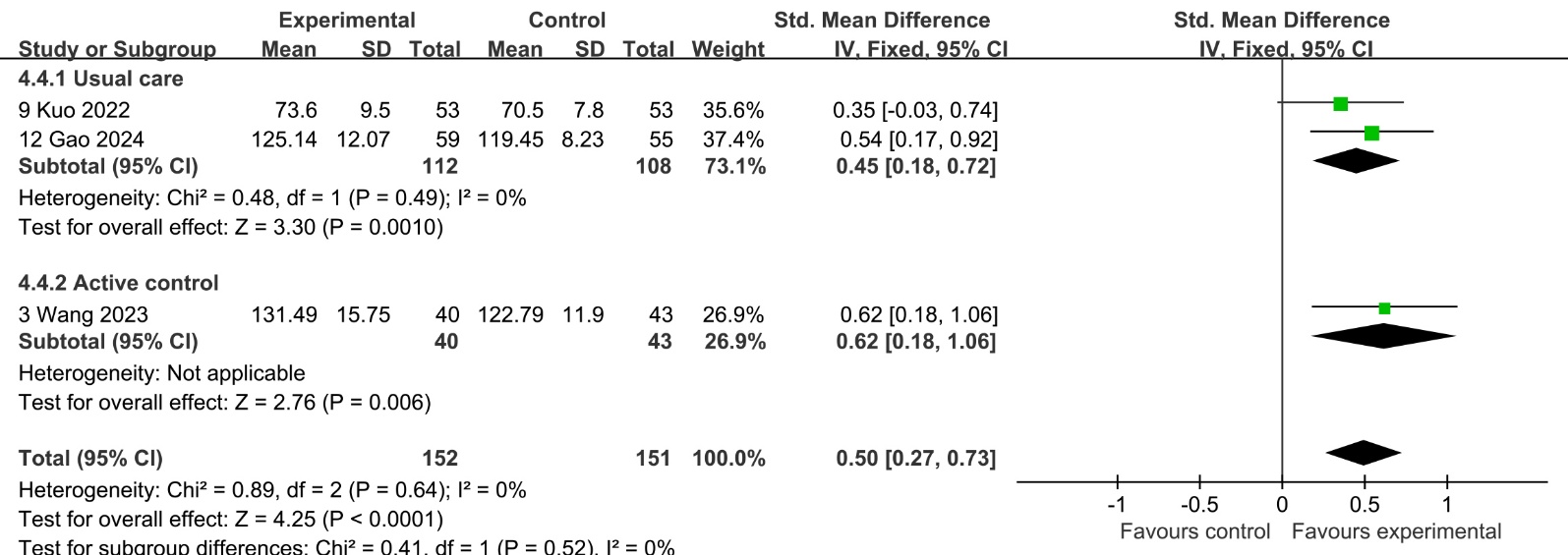


**4e. Subgroup analysis between studies applying usual care and active control: Caesarean section rate**


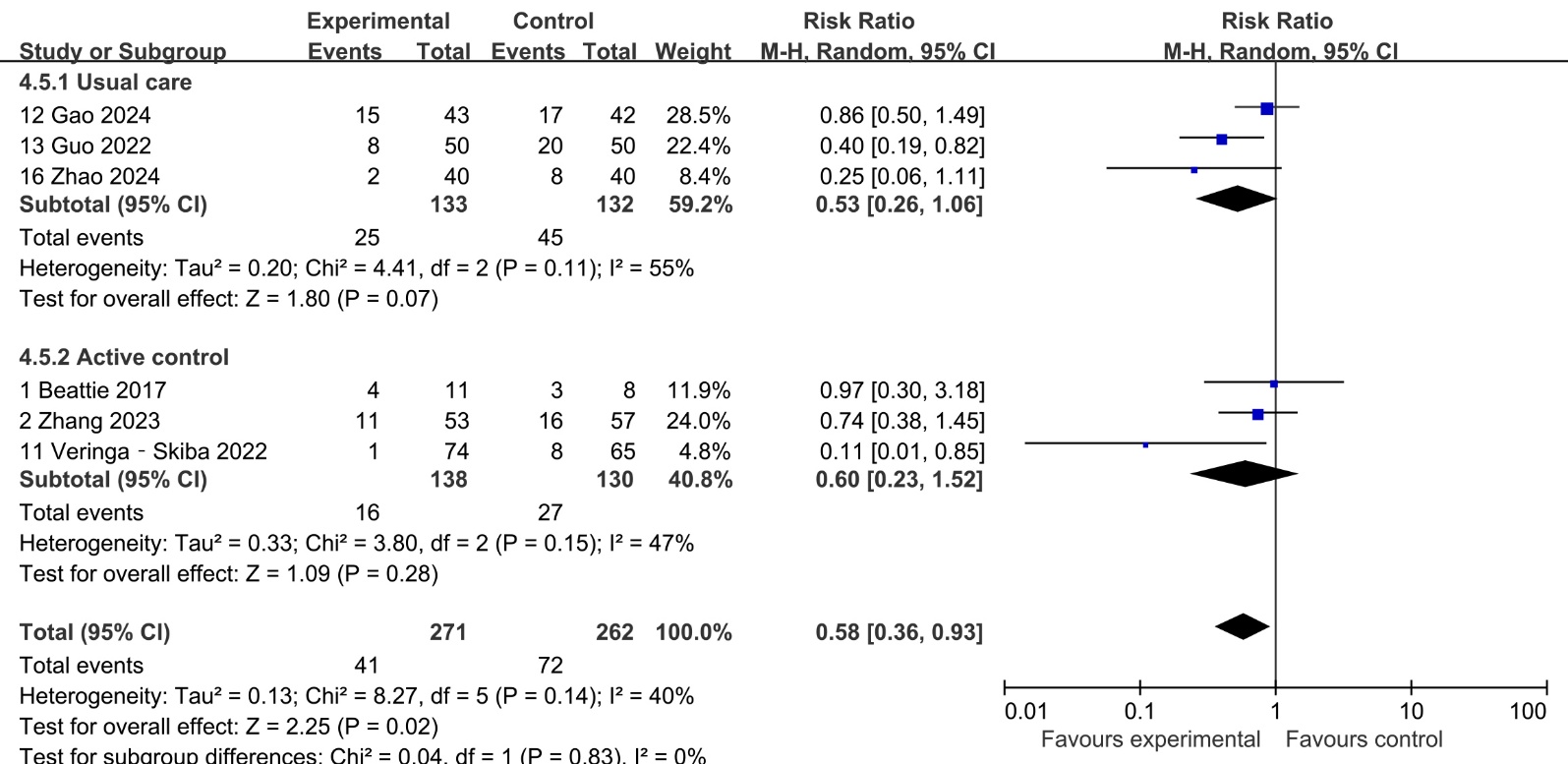


**4f. Subgroup analysis between studies applying usual care and active control: Epidural analgesia use rate**


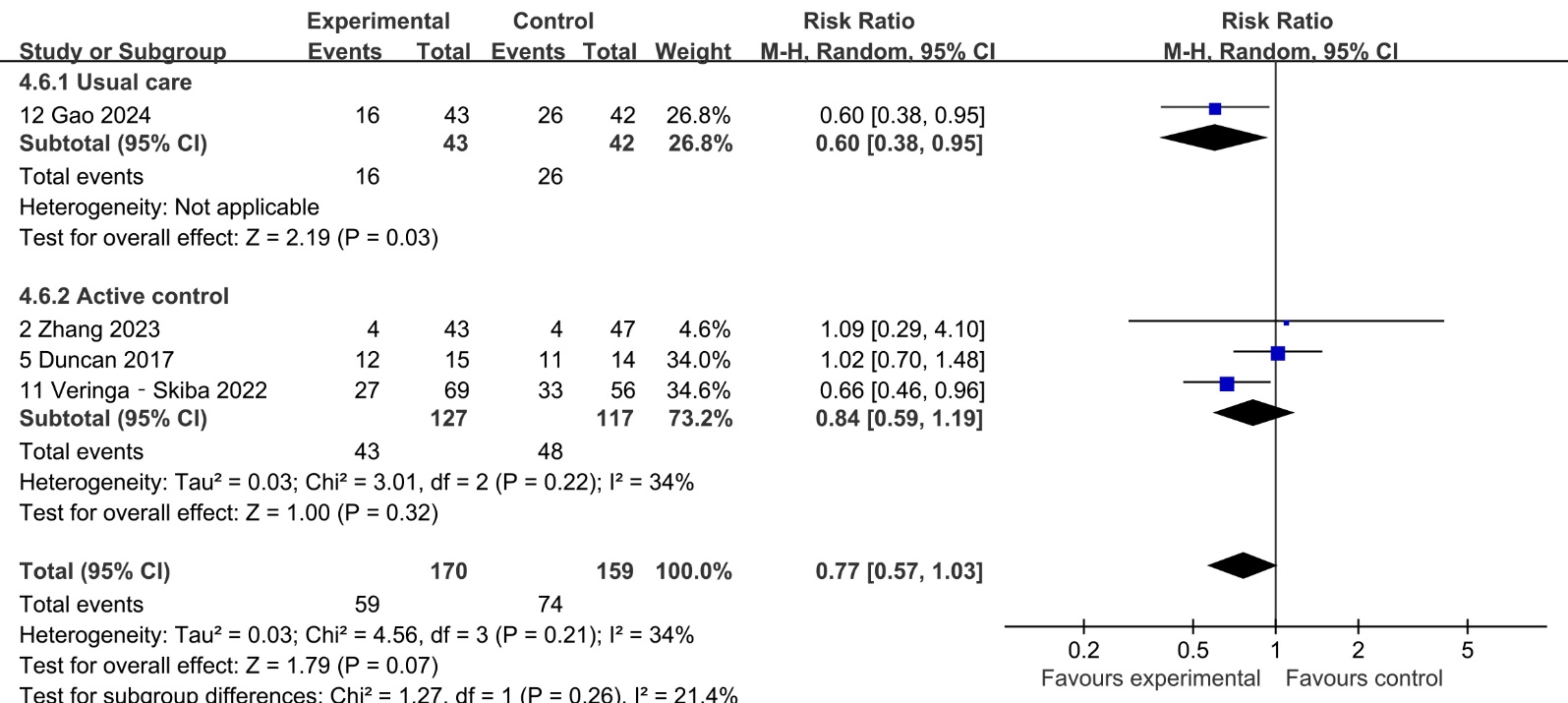

Supplement: Supplementary file 1 — Data S1: wvn70101‐sup‐0001‐Supinfo.docx. [file WVN-23-0-s001.docx]
